# Bacteroides salyersiae is a potent chondroitin sulfate-degrading species in the human gut microbiota

**DOI:** 10.1186/s40168-024-01768-2

**Published:** 2024-02-29

**Authors:** Yamin Wang, Mingfeng Ma, Wei Dai, Qingsen Shang, Guangli Yu

**Affiliations:** 1 Key Laboratory of Marine Drugs of Ministry of Education, Shandong Key Laboratory of Glycoscience and Glycotechnology, School of Medicine and Pharmacy, Ocean University of China, Qingdao, 266003 China; 2 Laboratory for Marine Drugs and Bioproducts, Laoshan Laboratory, Qingdao, 266237 China; 3 Qingdao Marine Biomedical Research Institute, Qingdao, 266071 China

**Keywords:** *Bacteroides salyersiae*, Chondroitin sulfate, Gut microbiota, *Bacteroides stercoris*, Glycosaminoglycans, Degradation, Fermentation, Short-chain fatty acids, Polysaccharides, Oligosaccharides

## Abstract

Chondroitin sulfate (CS) has widely been used as a symptomatic slow-acting drug or a dietary supplement for the treatment and prevention of osteoarthritis. However, CS could not be absorbed after oral intake due to its polyanionic nature and large molecular weight. Gut microbiota has recently been proposed to play a pivotal role in the metabolism of drugs and nutrients. Nonetheless, how CS is degraded by the human gut microbiota has not been fully characterized. In the present study, we demonstrated that each human gut microbiota was characterized with a unique capability for CS degradation. Degradation and fermentation of CS by the human gut microbiota produced significant amounts of unsaturated CS oligosaccharides (CSOSs) and short-chain fatty acids. To uncover which microbes were responsible for CS degradation, we isolated a total of 586 bacterial strains with a potential CS-degrading capability from 23 human fecal samples. *Bacteroides salyersiae* was a potent species for CS degradation in the human gut microbiota and produced the highest amount of CSOSs as compared to other well-recognized CS-degraders, including *Bacteroides finegoldii*, *Bacteroides thetaiotaomicron*, *Bacteroides xylanisolvens*, and *Bacteroides ovatus*. Genomic analysis suggested that *B. salyersiae* was armed with multiple carbohydrate-active enzymes that could potentially degrade CS into CSOSs. By using a spent medium assay, we further demonstrated that the unsaturated tetrasaccharide (udp4) produced by the primary degrader *B. salyersiae* could serve as a “public goods” molecule for the growth of *Bacteroides stercoris*, a secondary CS-degrader that was proficient at fermenting CSOSs but not CS. Taken together, our study provides insights into the metabolism of CS by the human gut microbiota, which has promising implications for the development of medical and nutritional therapies for osteoarthritis.

Video Abstract

**Supplementary Information:**

The online version contains supplementary material available at 10.1186/s40168-024-01768-2.

## Introduction

Osteoarthritis is a painful, disabling, and slowly developing degenerative disease that affects about 240 million people globally [1–3]. Chondroitin sulfate (CS) is a dominant family of sulfated linear polysaccharides that exist ubiquitously both on the cell surfaces and in the extracellular matrices of the human body [4–6]. Previous studies have indicated that CS is the most abundant structural component in the cartilage of the human joint tissues [4, 7, 8]. In this regard, following the recommendations of the European League Against Rheumatism (EULAR) and the European Society for Clinical and Economic Aspects of Osteoporosis, Osteoarthritis, and Musculoskeletal Diseases (ESCEO), CS has widely been used as a symptomatic slow-acting drug (SYSADOA) or a dietary supplement for the treatment and prevention of osteoarthritis [9, 10]. However, the use of CS for the management of osteoarthritis is still under debate since it cannot be absorbed after oral intake due to its polyanionic nature and large molecular weight [11–14]. Gut microbiota has recently been proposed to play a pivotal role in the metabolism of drugs and nutrients [15–17]. Nonetheless, how CS is degraded by the human gut microbiota has not been fully characterized [18].

## Results and discussion

**Fig. 1:**
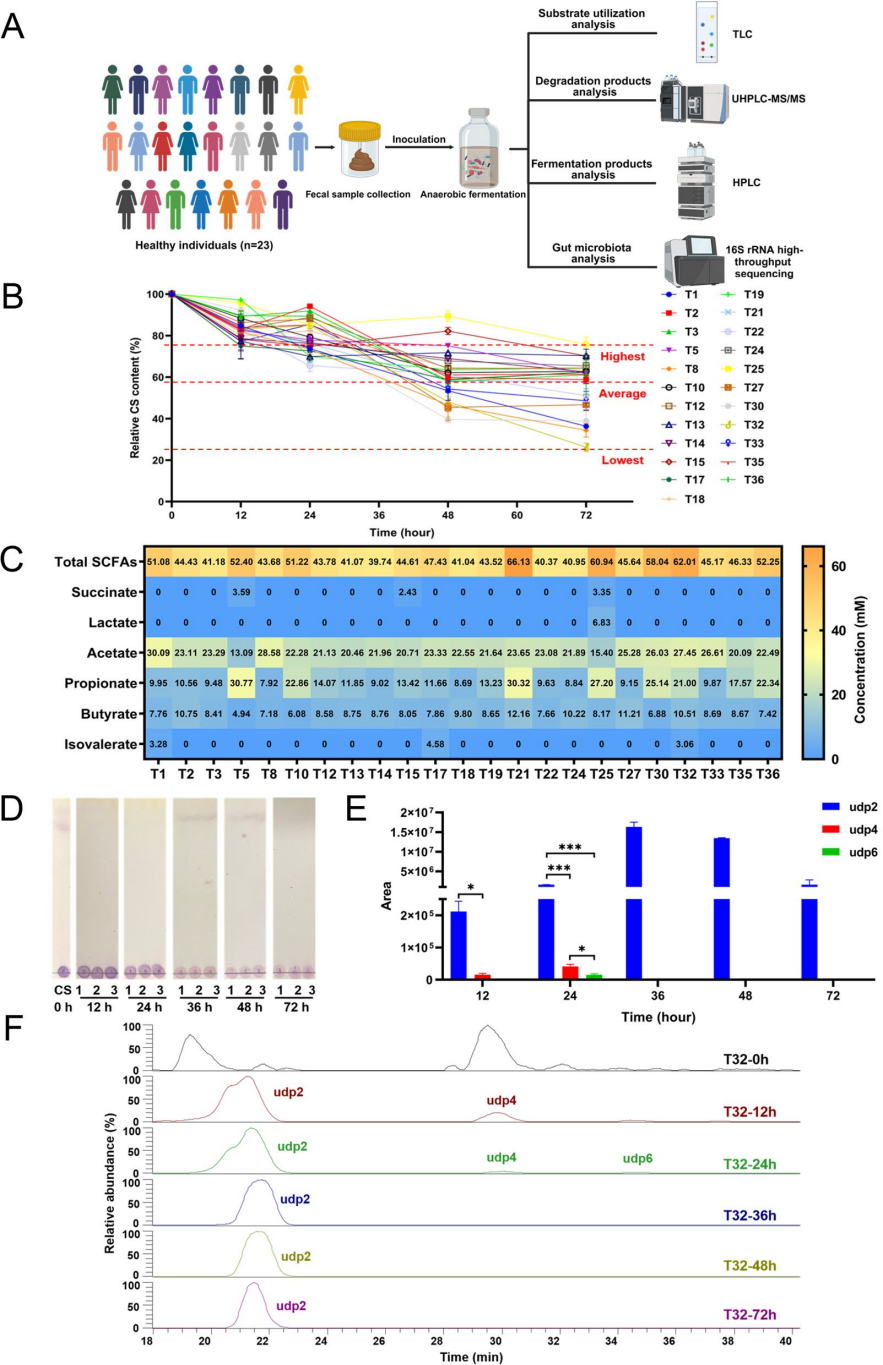
Degradation of CS by the human gut microbiota. Experimental design (**A**). Relative CS content in the culture medium (**B**). Heatmap of the concentrations of different SCFAs in the culture medium (**C**). TLC shows the degradation of CS by the gut microbiota of donor T32 (**D**). UPLC-MS/MS analysis of CSOSs in the culture medium of donor T32 (**E**). Total ion chromatograms showing the elution profiles of CSOSs in the culture medium of donor T32 at different time points (**F**). Part of the figure was created with BioRender.com. *p < 0.05; ***p < 0.001

To address this issue, we first investigated the degradation profiles of CS by the human gut microbiota from 23 individuals using in vitro anaerobic fermentation (Fig. 1A). We found that each human gut microbiota was characterized by a unique capability for CS degradation (Fig. 1B; Supplementary Figure S1 and S2). For example, the gut microbiota of donor T25 only utilized about 20% of the original CS while that of donor T32 utilized more than 70% of the original CS in the culture medium (Fig. 1B; Supplementary Figure S2). However, on average, the human gut microbiota degraded and fermented about 40% of the original CS within 48 h (Fig. 1B; Supplementary Figure S2). This suggested that CS was a readily degradable polysaccharide for most individuals’ gut microbiota and thus might be used as a microbiota-accessible carbohydrate (MAC).

Short-chain fatty acids (SCFAs) are major fermentation products of MACs in the human gut [19]. With the help of high-performance liquid chromatography (HPLC), we found that CS fermentation by the human gut microbiota produced a significant amount of SCFAs that were dominated by acetate, propionate, and butyrate (Fig. 1C). Although succinate, lactate, and isovalerate were also detected in the medium, they were produced at a much smaller amount (Fig. 1C). Thin layer chromatography (TLC) and ultra-performance liquid chromatography (UPLC)-mass spectrometry (MS)/MS further confirmed that CS was degraded by the human gut microbiota to produce a series of unsaturated CS oligosaccharides (CSOSs) with a degree of polymerization (dp) ranging from 2 to 8 (Fig. 1D–F; Supplementary Figure S2 and S3). However, it should be noted that although different CSOSs were produced at the very first 24 h, the tetrasaccharide (udp4), hexasaccharide (udp6), and octasaccharide (udp8) were further degraded by the gut microbiota to produce disaccharide (udp2) as the fermentation continued (Fig. 1E, F; Supplementary Figure S3). Moreover, udp2 was the only degradation product left in the culture medium after 36 h (Fig. 1E, F; Supplementary Figure S2 and S3).

Due to the polyanionic nature and large molecular weight, CS could not be directedly absorbed after oral intake [11–13]. However, previous human and animal studies have well demonstrated that CS could be absorbed in the form of udp2 after oral intake [11, 14]. In view of the above results, our study suggested that the gut microbiota might to some extent be able to contribute to the absorption of CS in the intestine by degrading it into oligosaccharides. However, more detailed studies are needed to verify this possibility.

**Fig. 2:**
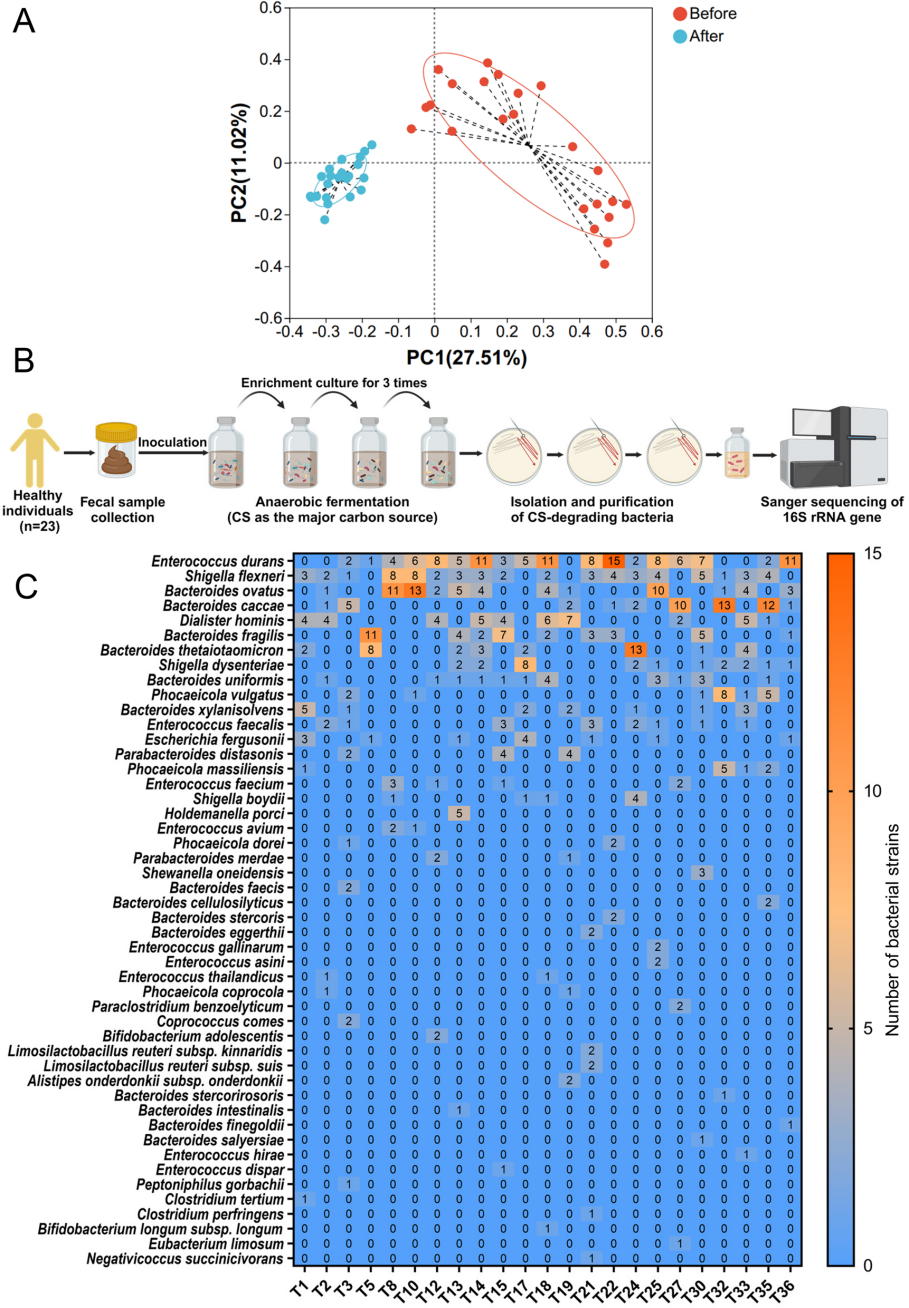
Isolation of CS-degrading bacteria from the human gut microbiota. Changes in the structure of the human gut microbiota before and after fermentation. PCA analysis (**A**). Experimental design for the isolation of CS-degrading bacteria from the human gut microbiota (**B**). Heatmap of the number of CS-degrading bacteria isolated from all 23 human fecal samples (**C**). Part of the figure was created with BioRender.com

Using 16S rRNA gene amplicon high-throughput sequencing and bioinformatics analysis, we found a significant change in the composition of the human gut microbiota before and after fermentation (Fig. 2A; Supplementary Figure S4). Besides, some genera were enriched upon cultivation in the medium containing CS as the major carbon source (Supplementary Figure S4 and S5), suggesting the possibility that they might have degraded and utilized CS as a substrate for their growth. We next wondered which bacteria were responsible for CS degradation in the human gut microbiota. To answer this question, we isolated a total of 586 bacterial strains with a potential CS-degrading capability from all 23 human fecal samples using the well-established enrichment culture method (Fig. 2B, C; Supplementary Figure S6 and Table S1). 16S rRNA gene-based phylogeny suggested that these fecal isolates belonged to 48 different species of bacteria (Fig. 2C; Supplementary Figure S7 and Table S1). This implied that the CS-degrading capability might be widely distributed among the human gut microbiota. Additionally, distinct species of bacteria were isolated from the fecal samples of different individuals (Fig. 2C; Supplementary Figure S6). This indicated that each individual was characterized by its own unique bacteria for CS degradation, a further argument for the individualized metabolism of CS by the human gut microbiota.

**Fig. 3:**
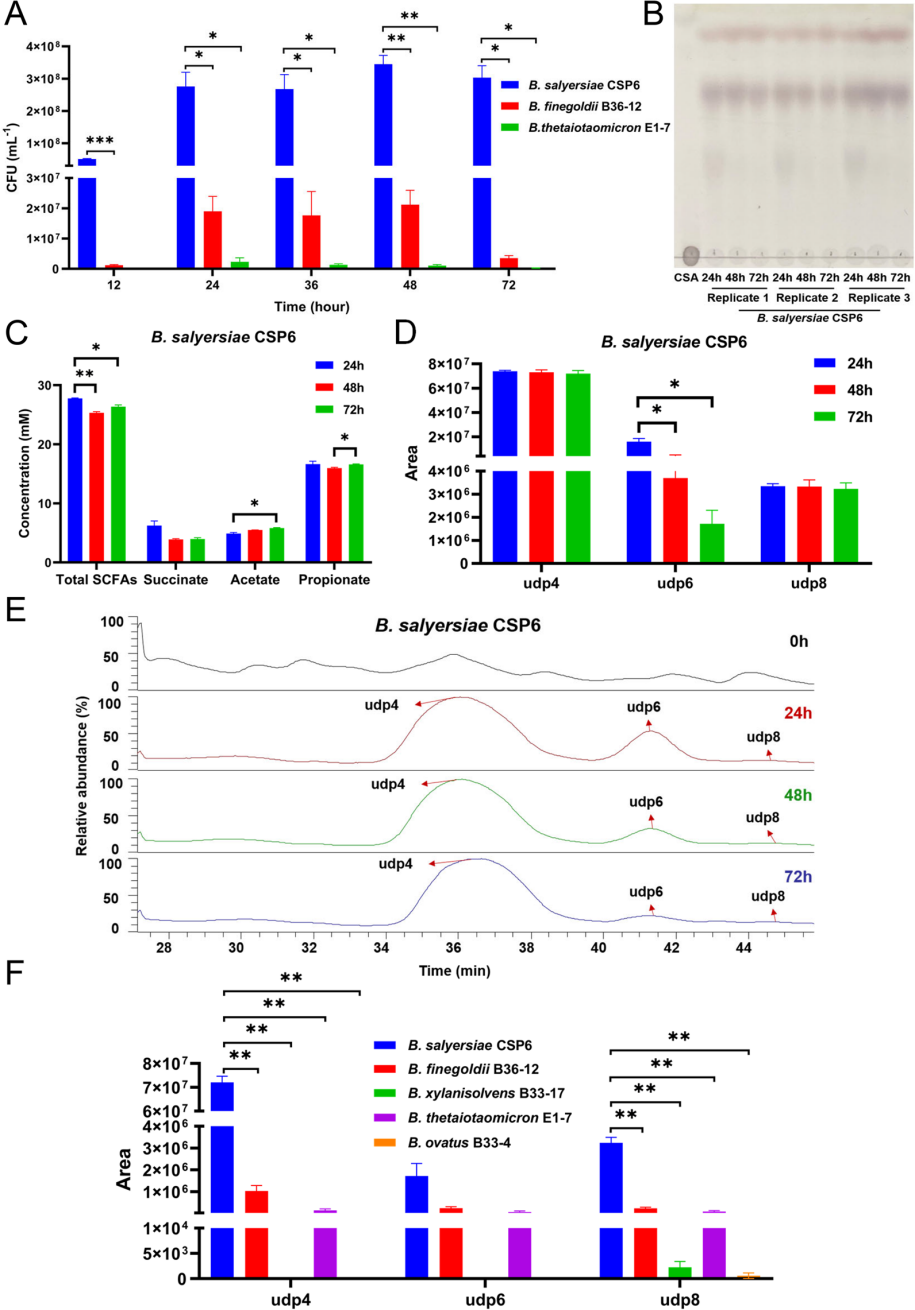
Degradation of CS by *B.* *salyersiae* CSP6. Colony forming units (CFUs) of *B.* *salyersiae* CSP6, *B.* *finegoldii* B36-12, and *B.* *thetaiotaomicron* E1-7 grew in the culture medium containing CS as the major carbon source (**A**). TLC showing the degradation of CS by *B.* *salyersiae* CSP6 (B). Concentrations of different SCFAs in the culture medium of *B.* *salyersiae* CSP6 (**C**). UPLC-MS/MS analysis of CSOSs produced by *B.* *salyersiae* CSP6 (**D**). Total ion chromatograms showing the elution profiles of CSOSs in the culture medium of *B.* *salyersiae* CSP6 at different time points (**E**). Comparison of the amount of CSOSs produced by *B.* *salyersiae* CSP6, *B.* *finegoldii* B36-12, *B.* *xylanisolvens* B33-17, *B.* *thetaiotaomicron* E1-7, and *B.* *ovatus* B33-4 at 72 h (**F**). *p < 0.05; **p < 0.01; ***p < 0.001

**Fig. 4:**
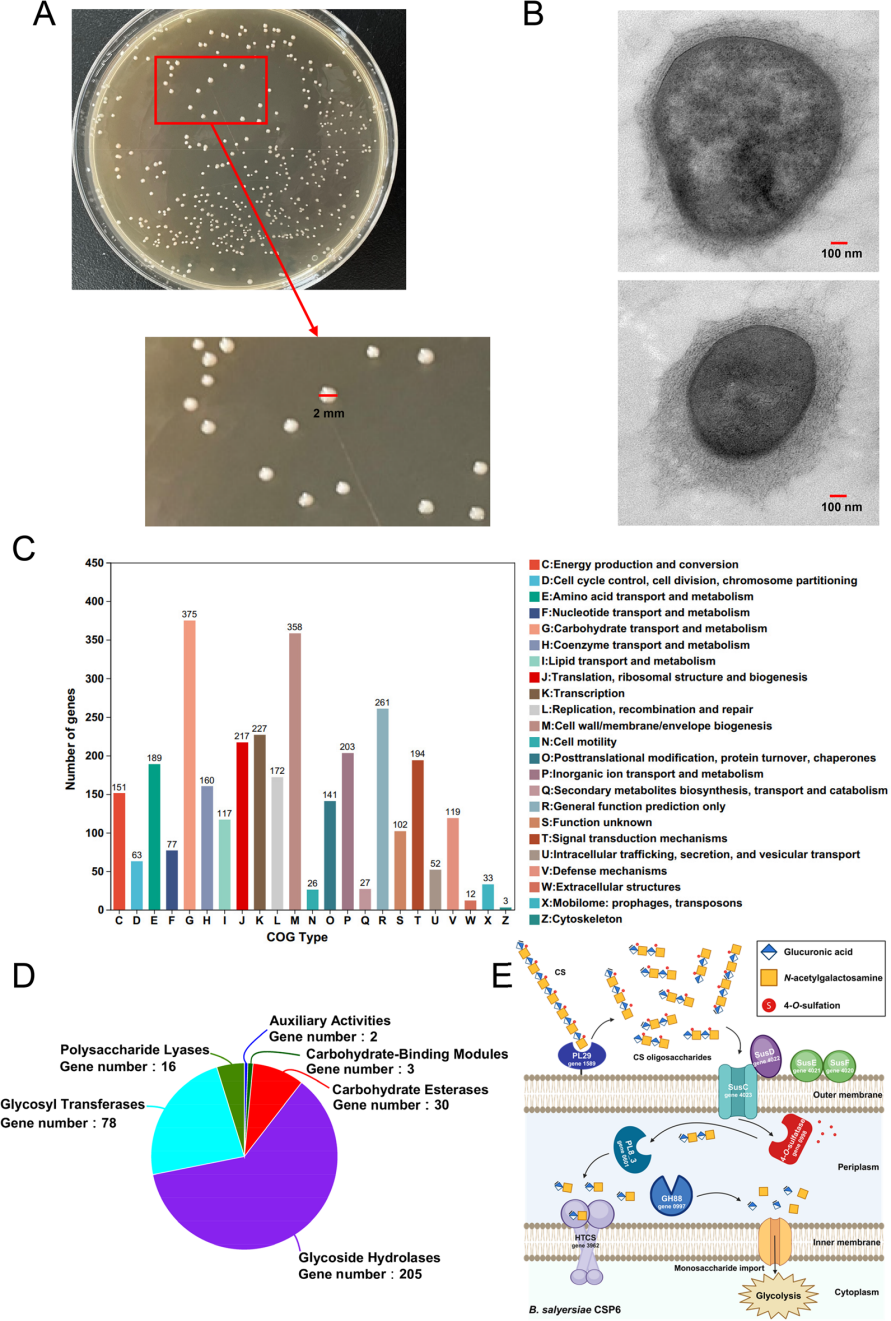
Cell morphology and genomic analysis of *B.* *salyersiae* CSP6. Colony morphology of *B.* *salyersiae* CSP6 on the plate (**A**). Transmission electron microscope (TEM) analysis of the cell morphology of *B.* *salyersiae* CSP6 (**B**). *B.* *salyersiae* CSP6 was a round-shaped bacterium with a cell size of about 800 to 1000 nm (diameter). COG function analysis of the genome of *B.* *salyersiae* CSP6 (**C**). Analysis of the CAZymes in the genome of *B.* *salyersiae* CSP6 (**D**). A proposed model for understanding the degradation of CS by *B.* *salyersiae* CSP6 based on genomic analysis (**E**). Part of the figure was created with BioRender.com

With all the 48 species of intestinal bacteria in hand, we next sought to investigate and compare their degrading capabilities. *Bacteroides salyersiae*, *Bacteoides finegoldii*, *Bacteroides xylanisolvens*, *Bacteroides thetaiotaomicron*, and *Bacteroides ovatus* were identified as top 5 degraders for CS in the human gut microbiota (Supplementary Figure S7). Preceding studies have shown that *B. finegoldii*, *B. xylanisolvens*, *B. thetaiotaomicron*, and *B. ovatus* are well-recognized CS-degraders in the human gut [20]. In line with previous results [17, 20], we found that these bacteria could degrade approximately 26 to 59% of the original CS within 72 h (Supplementary Figure S7). However, the amount of CS consumed by these bacteria was relatively smaller as compared to that consumed by *B. salyersiae*, a proficient CS-degrader identified in the present study (Fig. 3; Supplementary Figure S7–S9). Even in the very first 24 h, up to 85% of the original CS was successfully degraded by *B. salyersiae* (Supplementary Figure S7). This made *B. salyersiae* a potent CS-degrader within the gut microbiota of the 23 individuals. Besides, CS degradation by *B. salyersiae* produced the highest amount of CSOSs as compared to that produced by the four well-established CS-degraders (Fig. 3F; Supplementary Figure S10–S12). Additionally, *B. salyersiae* grew very well in both the liquid and solid culture medium containing CS as the major carbon source (Figs. 3A and 4A, B). The major degradation product produced by *B. salyersiae* was udp4 and the major fermentation product produced by *B. salyersiae* was propionate (Fig. 3B–E; Supplementary Figure S12).

To exclude the possibility that the CS-degrading capacity was specific to the particular isolate of *B. salyersiae* obtained in the present study, we further tested the CS-degrading capacity of another human gut bacterium *B. salyersiae* FL17. Although this strain was not isolated from the aforementioned 23 individuals using the enrichment culture method, it was found to have the same CS-degrading capacity as compared to the one isolated in the present study (Supplementary Figure S13). Both strains degraded CS to produce CSOSs in the culture medium (Supplementary Figure S13). Besides, both strains utilized an equal amount of CS during fermentation (Supplementary Figure S13). Altogether, our study suggested that the CS-degrading capacity of *B. salyersiae* identified in the present study was possibly a general characteristic of this species*.*

Given that *B. salyersiae* was a potent species for CS degradation in the 23 human fecal samples tested in our study, we then wondered how CS was degraded by this specific gut anaerobe. To answer this question, we sequenced the whole genome of *B. salyersiae*. Bioinformatics analysis suggested that the genome size of *B. salyersiae* was 5,561,372 bp and the G + C content in the genome was 41.95% (Supplementary Figure S14). Kyoto Encyclopedia of Genes and Genomes (KEGG) pathway analysis and clusters of orthologous groups (COG) function analysis indicated that most of the identified genes in *B. salyersiae* were involved in the carbohydrate transport and metabolism pathway (Fig. 4C; Supplementary Figure S14), suggesting that this bacterium might be skilled at degrading and metabolizing dietary polysaccharides. In this sense, we next analyzed the carbohydrate-active enzymes (CAZymes) in the genome of *B. salyersiae*. A total of 334 genes were identified as responsible for the expression of different classes of CAZymes including glycoside hydrolases (GHs), glycosyltransferases (GTs), polysaccharide lyases (PLs), carbohydrate esterases (CEs), carbohydrate-binding modules (CBMs), and auxiliary activities (AAs) in this bacterium (Fig. 4D).

The CAZymes that target the glycosidic bonds in CS from the *Bacteoides* spp. have been extensively studied [20]. Based on previous results [21–23], our genomic analysis suggested that PL29, PL8_3, and GH88 were candidate CS-metabolizing enzymes in *B. salyersiae* (Supplementary Table S2–S4). Besides, genes coding the starch utilization system (Sus) proteins were also identified in the genome of *B. salyersiae* (Supplementary Table S2–S4). The Sus proteins in *Bacteoides* spp. have been well-demonstrated to capture and transport different glycans [20, 23, 24]. Altogether, based on these bioinformatics results, we tentatively put forward a possible model for understanding the degradation of CS by *B. salyersiae* (Fig. 4E). However, although we have clearly demonstrated that CS was degraded by *B. salyersiae*, the CAZymes activities of this bacteria shown in the model have not been confirmed using genetic or biochemical approaches in the present research. Future studies are therefore warranted to further investigate the detailed molecular mechanisms involved in the degradation of CS by *B. salyersiae*.

**Fig. 5:**
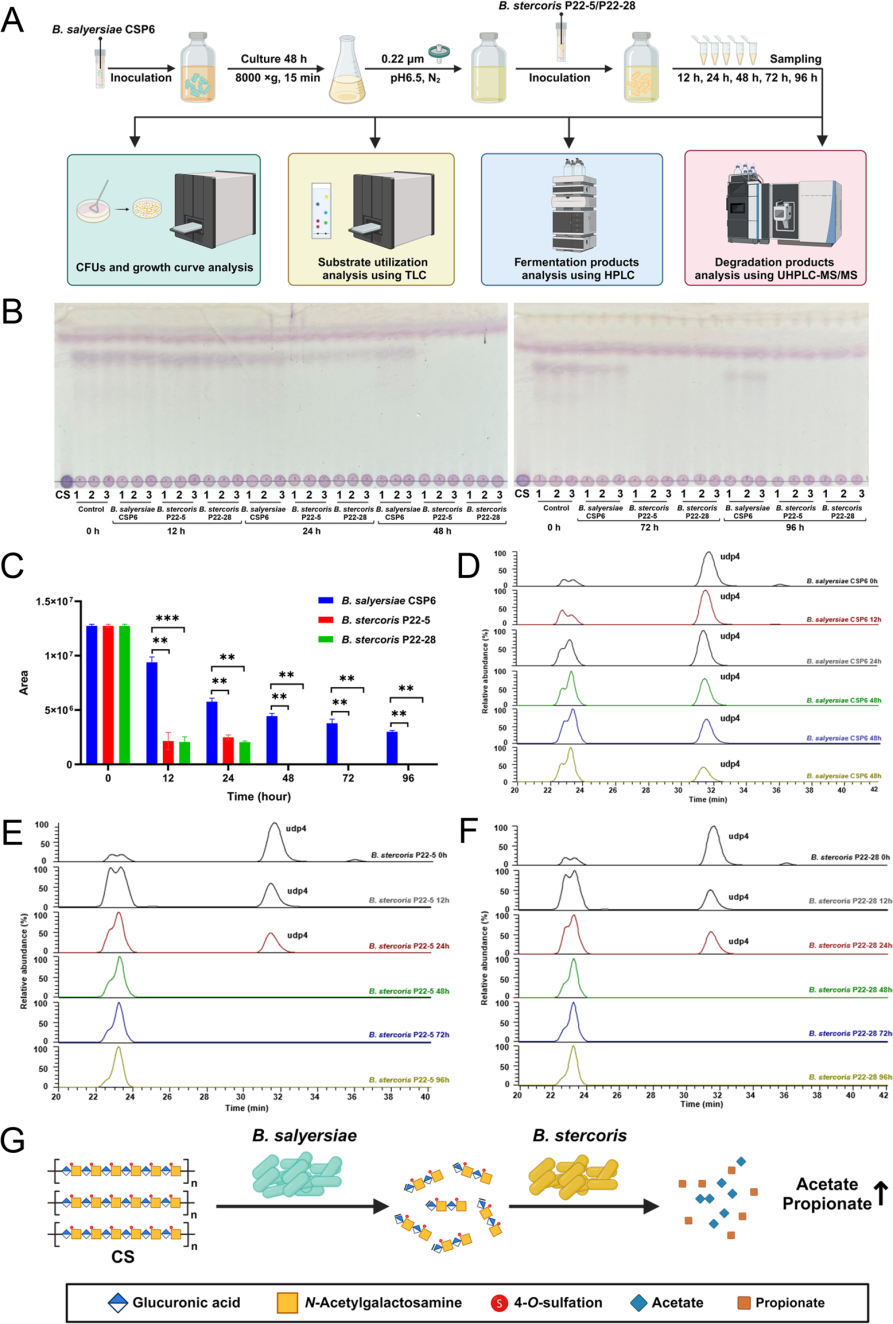
Cross-feeding interactions between *B.* *salyersiae* and *B.* *stercoris*. Experimental design of the spent medium assay (**A**). TLC showing the utilization of udp4 by *B.* *salyersiae* CSP6, *B.* *stercoris* P22-5, and *B.* *stercoris* P22-28 (**B**). *B.* *salyersiae* CSP6 was used as a negative control for the assay. The utilization of udp4 was monitored from 12 to 96 h using UPLC-MS/MS analysis. **C** Total ion chromatograms showing the elution profiles of udp4 in the culture medium of *B.* *salyersiae* CSP6 (**D**), *B.* *stercoris* P22-5 (**E**), and *B.* *stercoris* P22-28 (**F**) at different time points. A proposed model for understanding the cross-feeding interactions between *B.* *salyersiae* and *B.* *stercoris* (**G**). Part of the figure was created with BioRender.com. **p < 0.01; ***p < 0.001

As aforementioned, CS degradation by *B. salyersiae* produced a significant amount of udp4 in the culture medium (Fig. 3; Supplementary Figure S12). In this light, we next wondered if the produced udp4 could be further utilized by other anaerobes in the gut. To address this question, a spent medium assay was conducted to screen the candidate bacterium that could utilize the udp4 produced by *B. salyersiae* in the culture medium (Fig. 5A; Supplementary Figure S15). Of all the 54 different species of bacterial isolates tested, only *Bacteroides stercoris* was found to have the capability for udp4 utilization (Fig. 5; Supplementary Figure S16). This suggested that the udp4-utilizing capability might be very specific to only a few species of anaerobes in the human gut.

*B. stercoris* utilized about 40% of udp4 in the spent medium within 48 h (Fig. 5B–F; Supplementary Figure S16 and Figure S17). Besides, fermentation of udp4 by *B. stercoris* produced significant amounts of acetate and propionate (Supplementary Figure S16). Nonetheless, it should be noted that *B. stercoris* itself was not a good CS-degrader as it only degraded about 20% of the original CS in the culture medium even after 72 h (Supplementary Figure S7). Collectively, these results indicated that the udp4 produced by the primary degrader *B. salyersiae* had the potential to serve as a “public goods” molecule for the growth of *B. stercoris*, a secondary CS-degrader that was skilled at utilizing CSOSs but not CS (Fig. 5G).

Our study does not rule out the possibility that other CSOSs such as udp2, udp6, and udp8 might also be able to mediate the cross-feeding interactions between the primary and secondary CS-degraders in the human intestine. This could be the subject of future research. The ability of *B. stercoris* to utilize udp4 produced by *B. salyersiae* suggested that some bacteria might have co-evolved to work synergistically to degrade CS in the human gut. The cross-feeding interactions between *B. salyersiae* and *B. stercoris* identified in the present study provided a framework for understating the degradation and metabolism of CS in the human gut.

The evolution of the metabolic cross-feeding interactions between specific bacteria in the human intestine has been proposed to be driven by different factors [25–27]. Our study demonstrated that the udp4 produced by the primary degrader during the metabolism of CS could serve as a “public goods” nutrient for the growth of the secondary degrader in the same niche. In accordance with previous results [28–30], our findings reinforced the notion that intermediate oligosaccharides produced during the degradation of complex carbohydrates had the potential to drive the multi-species symbiotic cross-feeding in the human gut. These results opened a new window for understanding the modulatory effect of CS on the human gut microbiota. This is critically important since in vivo studies have well demonstrated that the gut microbiota is deeply involved in the pathogenesis of osteoarthritis [31–33] and that changes in the composition of gut microbiota are determining factors for achieving the therapeutic effect of CS in vivo [34–36].

## Conclusions

**Fig. 6:**
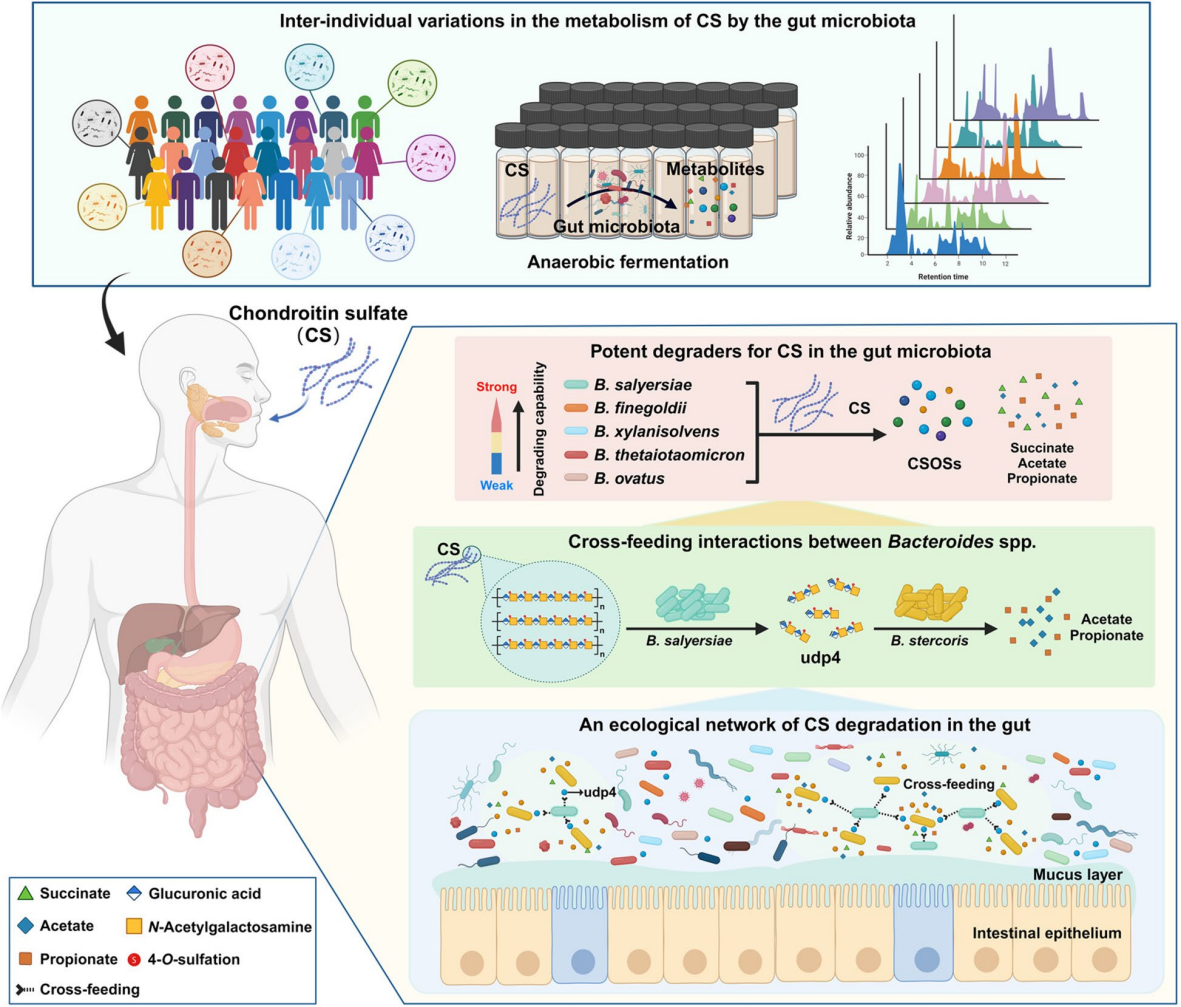
A schematic diagram illustrating the degradation of CS by the human gut microbiota. Each gut microbiota was characterized by a unique capability for CS degradation. CS was readily degraded and fermented by specific anaerobes in the gut to produce SCFAs and CSOSs. These bacteria included *B.* *salyersiae*, *B.* *finegoldii*, *B.* *xylanisolvens*, *B.* *thetaiotaomicron*, and *B.* *ovatus*. *B.* *salyersiae* was identified as a potent bacterium for CS degradation in the present study. The udp4 produced by the primary degrader *B.* *salyersiae* sustained the growth of the secondary degrader *B.* *stercoris*. *B.* *salyersiae* and *B.* *stercoris* might have co-evolved to work synergistically to degrade and utilize CS in the human gut. The figure was created with BioRender.com

Taken together, in the present research, we comprehensively investigated the detailed degradation profiles of CS by the gut microbiota from 23 healthy individuals and illustrated that *B. salyersiae* was a potent species for CS degradation in the human intestinal microbiota (Fig. 6). Our study suggested that *B. salyersiae* was potentially a keystone species for CS degradation in the human intestine. The udp4-based cross-feeding interactions identified in our study provide insights into the metabolism of CS by the human gut microbiota, which has promising implications for the development of medical and nutritional therapies for osteoarthritis.

### Supplementary Information

**Additional file 1: Figure S1.** TLC showing the degradation of CS by the human gut microbiota. The degradation was monitored at 12 hours (A), 24 hours (B), 36 hours (C), 48 hours (D), and 72 hours (E). **Figure S2.** Degradation of CS by the human gut microbiota. Relative CS content in the culture medium at 72 hours (A). UPLC-MS/MS analysis of CSOSs in the culture medium of donor T25 (B). Total ion chromatograms showing the elution profiles of CSOSs in the culture medium of donor T25 at different time points (C). **Figure S3.** Mass spectrum showing the signals of udp2 (A), udp4 (B), and udp6 (C) according to their m/z ratios. The CSOSs, including udp2, udp4, and udp6 were produced in the culture medium as a result of CS degradation by the human gut microbiota. **Figure S4.** Changes in the structure of the human gut microbiota before and after fermentation. Venn diagram showing the differences of the operational taxonomic units (OTUs) (A). Observed species (B). Chao1 index (C). Shannon index (D). Heatmap of the abundance of gut bacteria at the genus level (E). **Figure S5.** Differences in the composition of the human gut microbiota before and after fermentation. Wilcoxon rank-sum test analysis of the gut microbiota at the species level (A). Linear discriminant analysis (LDA) Effect Size (LEfSe) analysis of the gut microbiota at the species level (B). Only bacterial taxa with an LDA score of above 3.0 were listed. **Figure S6.** Isolation of CS-degrading bacteria from the human gut microbiota. Different species of bacteria were obtained from different human fecal samples (A-W). **Figure S7.** *B.* *salyersiae* CSP6 was identified as a potent bacterium for CS-degradation in the present study. Heatmap of the relative abundance of the consumed CS (A). Phylogenetic tree analysis of the CS-degrading bacteria based on the 16S rRNA gene (B). **Figure S8.** TLC showing the degradation of CS by different human fecal isolates. The results were presented from *B.* *thetaiotaomicron* E1-7 to *H.* *porci* E13-26 (A-I). **Figure S9.** TLC showing the degradation of CS by different human fecal isolates. The results were presented from *E.* *durans* E13-16 to *S.* *oneidensis* P30-2-30 (A-H). **Figure S10.** Degradation and fermentation of CS by *B.* *finegoldii* B36-12, *B.* *thetaiotaomicron* E1-7, *B.* *xylanisolvens* B33-17, and *B.* *ovatus* B33-4. Concentrations of different SCFAs in the culture medium of *B.* *finegoldii* B36-12 (A), *B.* *thetaiotaomicron* E1-7 (B), *B.* *xylanisolvens* B33-17 (C), and *B.* *ovatus* B33-4 (D). * *p* < 0.05; ** *p* < 0.01. **Figure S11.** CS degradation by *B.* *finegoldii* B36-12, *B.* *thetaiotaomicron* E1-7, *B.* *xylanisolvens* B33-17, and *B.* *ovatus* B33-4. UPLC-MS/MS analysis of CSOSs produced by *B.* *finegoldii* B36-12 (A), *B.* *thetaiotaomicron* E1-7 (B), *B.* *xylanisolvens* B33-17 (C), and *B.* *ovatus* B33-4 (D). Total ion chromatograms showing the elution profiles of CSOSs in the culture medium of *B.* *finegoldii* B36-12 (E), *B.* *thetaiotaomicron* E1-7 (F), *B.* *xylanisolvens* B33-17 (G), and *B.* *ovatus* B33-4 (H) at different time points. * *p* < 0.05. **Figure S12.** Mass spectrum showing the signals of udp4 (A), udp6 (B), and udp8 (C) according to their m/z ratios. The CSOSs, including udp4, udp6, and udp8 were produced in the culture medium as a result of CS degradation by *B.* *salyersiae* CSP6, *B.* *finegoldii* B36-12, *B.* *xylanisolvens* B33-17, *B.* *thetaiotaomicron* E1-7, and *B.* *ovatus* B33-4. **Figure S13.** Degradation of CS by different strains of *B.* *salyersiae*. TLC showing the degradation of CS by *B.* *salyersiae* CSP6 and *B.* *salyersiae* FL17 (A). Relative carbohydrate content in the culture medium at different time points (B). *B.* *salyersiae* FL17 was previously isolated from the fecal sample of a healthy individual. This individual has not participated in the present study. **Figure S14.** Genome analysis of *B.* *salyersiae* CSP6. COG function classification (A). KEGG pathway analysis (B). **Figure S15.** Screening of candidate bacteria that could utilize udp4 using the spent medium assay. TLC showing the utilization of udp4 by different human gut bacteria. (A). List of the tested bacteria (B). **Figure S16.** Cross-feeding interactions between *B.* *salyersiae* and *B.* *stercoris* identified using the spent medium assay. Relative carbohydrate content in the culture medium (A). Growth curve (B) and CFU analysis (C). Concentrations of total SCFAs (D), acetate (E), and propionate (F) in the culture medium of *B.* *salyersiae* and *B.* *stercoris*. * *p* < 0.05; ** *p* < 0.01; *** *p* < 0.001. **Figure S17.** Mass spectrum showing the signal of udp4 according to the m/z ratio. The udp4 concentration in the spent medium was analyzed using UPLC-MS/MS. **Table S1.** Summary of CS-degrading bacteria isolated from the human fecal samples. **Table S2.** Summary of the potential enzymes for CS degradation in *B.* *salyersiae* CSP6 based on the genomic analysis. **Supplementary Table S3.** Genome annotation of B. salyersiae CSP6. **Supplementary Table S4.** CAZyme annotation of B. salyersiae CSP6.

## Data Availability

The whole genome sequence of *B. salyersiae* CSP6 was deposited in the GenBank under the accession number CP133452. The BioProject and BioSample accession numbers were PRJNA1007784 and SAMN37098068, respectively. The high-throughput sequencing data of the 16S rRNA gene amplicons of the human gut microbiota before and after fermentation was deposited in the GenBank under the BioProject number PRJNA1011813.

## Abbreviations

CS: Chondroitin sulfate
SCFAs: Short-chain fatty acids
CSOSs: Chondroitin sulfate oligosaccharides
dp: Degree of polymerization
udp2: Unsaturated disaccharide
udp4: Unsaturated tetrasaccharide
udp6: Unsaturated hexasaccharide
udp8: Unsaturated octasaccharide
CAZymes: Carbohydrate-active enzymes
PLs: Polysaccharide lyases
GHs: Glycoside hydrolases
CEs: Carbohydrate esterases
GTs: Glycosyltransferases
CBMs: Carbohydrate-binding modules
AAs: Auxiliary activities
Sus: Starch utilization system
PULs: Polysaccharide utilization loci
TLC: Thin layer chromatography
HPLC: High-performance liquid chromatography
UPLC: Ultra-performance liquid chromatography
MS: Mass spectrometry
KEGG: Kyoto Encyclopedia of Genes and Genomes
COG: Clusters of orthologous groups
TEM: Transmission electron microscope
SYSADOA: Symptomatic slow-acting drug for osteoarthritis
EULAR: European League Against Rheumatism
ESCEO: European Society for Clinical and Economic Aspects of Osteoporosis, Osteoarthritis and Musculoskeletal Diseases
MAC: Microbiota-accessible carbohydrate
CFUs: Colony forming units
OD: Optical density
