# Supplementary material for: Bacteroides salyersiae is a potent chondroitin sulfate-degrading species in the human gut microbiota
Source: Microbiome. 2024 Feb 29;12:41. doi: 10.1186/s40168-024-01768-2 (PMC10902947; doi:10.1186/s40168-024-01768-2)

***Bacteroides salyersiae* is a potent chondroitin sulfate-degrading species in the human gut microbiota**

Yamin Wang ^1^, Mingfeng Ma ^1^, Wei Dai ^1^, Qingsen Shang ^1, 2, 3 *^, Guangli Yu ^1, 2 *^

*1 Key Laboratory of Marine Drugs of Ministry of Education, Shandong Key Laboratory of Glycoscience and Glycotechnology, School of Medicine and Pharmacy, Ocean University of China, Qingdao 266003, China;*

*2 Laboratory for Marine Drugs and Bioproducts, Laoshan Laboratory, Qingdao 266237, China;*

*3 Qingdao Marine Biomedical Research Institute, Qingdao, 266071, China;*

*Corresponding authors:

Qingsen Shang, School of Medicine and Pharmacy, Ocean University of China, Qingdao, 266003, China; E-Mail: shangqingsen@ouc.edu.cn; shangqingsen@163.com

Guangli Yu, School of Medicine and Pharmacy, Ocean University of China, Qingdao, 266003, China; E-Mail: glyu@ouc.edu.cn

**1. Supplementary Methods and Materials**

**1.1 Chemicals and reagents**

The standard solution of the short-chain fatty acids (SCFAs), including lactate, acetate, propionate, succinate, isovalerate, and butyrate were all purchased from the Sigma-Aldrich (St. Louis, MO, USA). Tryptone, peptone, yeast extract, and Tween 80 used for the *in vitro* anaerobic fermentation experiments were also obtained from the Sigma-Aldrich (St. Louis, MO, USA). Hemin chloride, formic acid, *n*-butanol, orcinol, glucose, chondroitin sulfate (CS), agar, and L-cysteine hydrochloride were all acquired from the Sangon Biotech (Shanghai, China).

Phosphate-buffered saline (PBS) was obtained from the Servicebio Technology (Wuhan, Hubei, China). The purified water used for medium preparation and chromatography analysis was purchased from the Wahaha Group (Hangzhou, Zhejiang, China). The purified acetonitrile used for the liquid chromatography was obtained from the Merck KGaA (Darmstadt, Germany). All other analytical grade chemicals, including NaCl, KCl, KH_2_PO_4_, MgSO_4_·7H_2_O, CaCl_2_·2H_2_O, MnCl_2_·4H_2_O, FeSO_4_·7H_2_O, CoCl_2_·H_2_O, ZnSO_4_·7H_2_O, CuSO_4_·5H_2_O, NiCl_2_·6H_2_O, concentrated sulfuric acid, phenol, ethanol, ethyl acetate, diphenylamine, aniline, phosphoric acid, concentrated hydrochloric acid, and acetic acid used in the present study were purchased from the Sinopharm Chemical (Shanghai, China).

**1.2 Degradation of CS by the human gut microbiota**

The fresh fecal samples were collected from 23 healthy volunteers (aged 20-58 years) who resided in Qingdao (Shandong, China). All individuals had not received any antibiotics, prebiotics, probiotics or postbiotics for at least one year prior to the sample collection. All individuals provided a signed consent for their participation. The human experiments for the collection of fecal samples were approved and supported by the Ethical Committee of the Ocean University of China, School of Medicine and Pharmacy (Permission No. OUC-2021-1011-01). The collection of the human fecal samples was conducted according to the International Committee of Medical Journal Editors (ICMJE) guidelines on Protection of Research Participants.

The fresh fecal samples were carefully collected into 50 mL sterile tubes and sealed within 3 minutes of defecation. The tubes were sterilized using a GI54DWS autoclave from the Zealway Instrument (Xiamen, Fujian, China) at 121 °C for 20 minutes. After that, the samples were immediately transferred to an Electrotek AW 500SG anaerobic (80% N_2_, 10% H_2_, and 10% CO_2_) chamber (Shipley, West Yorkshire, UK). Then, about 10 grams of the human fecal sample were homogenized with sterile PBS to prepare a 20% (wt/vol) slurry in the chamber. Food residues in the fecal samples were carefully removed by slowly passing the slurries through a 0.4 mm sieve. After that, 1 mL of the obtained slurry was inoculated into 50 mL of the culture medium to start the fermentation.

The well-established VI medium was applied to investigate the CS-degrading capabilities of the human gut microbiota with some modifications [1-3]. CS was added to the VI medium as the major carbon source as previously described [3]. The concentrations of different nutrients in the VI medium were as follows: CS, 8.0 g/L; tryptone, 3.0 g/L; peptone, 3.0 g/L; yeast extract, 4.5 g/L; L-cysteine hydrochloride, 0.8 g/L; hemin chloride, 0.05 g/L; NaCl, 4.5 g/L; KCl, 2.5 g/L; MgSO_4_·7H_2_O, 4.5 g/L; CaCl_2_·6H_2_O, 0.2 g/L; KH_2_PO_4_, 0.4 g/L; tween 80, 1 mL/L; trace elements solution, 0.2 mL/L. The trace elements solution used in the present study contained the following chemicals: MgSO_4_·7H_2_O, 3.0 g/L; CaCl_2_·2H_2_O, 0.1 g/L; MnCl_2_·4H_2_O, 0.32 g/L; FeSO_4_·7H_2_O, 0.1 g/L; CoCl_2_·H_2_O, 0.18 g/L; ZnSO_4_·7H_2_O, 0.18 g/L; CuSO_4_·5H_2_O, 0.01 g/L; NiCl_2_·6H_2_O, 0.092 g/L.

The fermentation experiments were carried out anaerobically (80% N_2_, 10% H_2_ and 10% CO_2_) at 37 °C in the aforementioned Electrotek AW 500SG anaerobic chamber (Shipley, West Yorkshire, UK). All the fermentation experiments were conducted with three biological replicates (n=3). During the fermentation process, about 5 mL of the culture medium was collected at different time points (12 hours, 24 hours, 48 hours, and 72 hours) to check the degradation of CS by the human gut microbiota. The medium was centrifugated at 8,000 *g* for 15 minutes to remove the bacteria and other insoluble materials using a Beckman Coulter Microfuge 20 benchtop centrifuge (Brea, CA, USA). The obtained medium was stored at -20 °C before subsequent analysis.

**1.3 SCFAs analysis**

The SCFAs produced during fermentation were analyzed using the well-established high-performance liquid chromatography (HPLC) (Agilent 1260, Santa Clara, US) method [1, 2]. Briefly, 1 mL of the fermentation medium was first acidified with equal amount of 1% (vol/vol) sulfuric acid. Then, 20 μL of the obtained medium was analyzed by HPLC after filtering through a MF-Millipore 0.22 µm membrane (Merck, Darmstadt, Germany).

An Aminex HPX-87H ion-exclusion column (Bio-Rad, Hercules, USA) coupled with an ultraviolet (UV) detector set at a wavelength of 210 nm was used for the SCFAs analysis. The HPLC system was isocratically run using a 5 mM sulfuric acid solution as the mobile phase at 50 °C. The flow rate of the mobile phase was set at 0.6 mL/min during the analysis. A series of standard SCFAs solutions (5 mM, 10 mM, 20 mM, 40 mM, 50 mM, 75 mM, and 100 mM) were prepared and used to obtain a calibration plot. The calibration plot was generated using averaged values. All the samples were analyzed in a randomized order.

**1.4 Total carbohydrate analysis**

The concentrations of CS and unsaturated tetrasaccharide (udp4) in the culture media were determined using the well-established phenol-sulfuric acid method as previously described [1-4]. This method was a simple and rapid colorimetric approach for the analysis of the total carbohydrates in the culture medium. The 80% (wt/wt) phenol solution was prepared by adding 20 g purified water to 80 g of redistilled phenol. The glucose solution (100 mg/L) was used as a standard for the analysis. The breakdown products of the carbohydrate reacted with phenol to produce a yellow-gold color. The absorbance of the resulting solution was measured spectrophotometrically at a wavelength of 490 nm using a Tecan Spark 10M microplate spectrophotometer (Untersbergstr, Grödig, Austria).

**1.5 Thin-layer chromatography (TLC) analysis**

TLC analysis was carried out to monitor and investigate the degradation of CS by the human gut microbiota. Briefly, the collected culture medium was first filtered using a MF-Millipore 0.22 µm membrane (Merck, Darmstadt, Germany). After that, a 0.6 μL aliquot of the medium was carefully loaded onto a pre-coated silica gel-60 aluminum TLC plate (Merck, Darmstadt, Germany). The samples were resolved using the freshly prepared formic acid/n-butanol/water (6:4:1, vol/vol/vol) solution as an eluent. After that, the loaded plate was immersed into the orcinol-sulfuric acid reagent or diphenylamine reagent. Then, the carbohydrate was visualized by heating the TLC plate for about 2 to 3 minutes at 105 °C in a DHG-9053A oven from the Yiheng Scientific Instruments (Shanghai, China).

The orcinol-sulfuric acid reagent was prepared by dissolving 0.9 g of orcinol in 25 mL of purified water and then mixing it with 375 mL of ethanol. After that, 50 mL of pre-cooled (4 °C) concentrated sulfuric acid was added very slowly to the obtained solution on ice. The reagent should be stored at 4 °C and be protected from light. The reagent should be used within one week. The diphenylamine reagent was prepared by dissolving 4.0 g of diphenylamine in 200 mL of ethyl acetate and 4 mL of aniline. After that, 20 mL of pre-cooled (4 °C) 85% (vol/vol) phosphoric acid and 2 mL of concentrated hydrochloric acid was added to the solution. The reagent was prepared by stirring the obtained solution overnight. The reagent should also be stored at 4 °C and be protected from light. Similarly, the reagent should also be used within 3 months. Both the orcinol-sulfuric acid reagent and the diphenylamine reagent are widely used for carbohydrate analysis in the TLC experiment. Generally, the orcinol-sulfuric acid reagent is less toxic and harmful while the diphenylamine reagent is more stable.

The diphenylamine reagent was specifically used for the TLC analysis and total carbohydrate analysis of CS degradation by the primary degraders isolated from human fecal samples. This was because the aforementioned phenol-sulfuric acid method used for the quantification of CS in the culture medium did not work for these primary degraders. Part of the reason was that these bacteria produced a significant amount of unsaturated CS oligosaccharides (CSOSs) in the media. In this regard, to investigate the CS degradation by these fecal isolates, we turned to analyze the gray value of the CS spot in the TLC image using the ImageJ software (National Institutes of Health, USA). This method for the carbohydrate analysis in the culture medium has been validated in our previous study [5]. The diphenylamine reagent was chosen for the analysis in our study because we found that the TLC image visualized using this reagent was clearer than that obtained using the orcinol-sulfuric acid reagent.

**1.6 UPLC-MS/MS analysis**

UPLC-MS/MS analysis for the quantification of the unsaturated CS oligosaccharides (CSOSs) produced during CS degradation by the human gut microbiota or consumed by the fecal isolates in the spent medium assay was performed using the methods previously developed in our lab [2, 5, 6]. The Agilent 1290 LC ultra-performance liquid chromatography (UPLC) system (Wilmington, DE, USA) equipped with an Thermo Fisher Scientific LTQ Orbitrap XL mass spectrometer (MS) (Waltham, MA, USA) was applied for the analysis.

The CSOSs were first isolated and purified from the culture medium using a Thermo Fisher Scientific HyperSep Hypercarb SPE column (Waltham, MA, USA). After that, the obtained CSOSs were concentrated and lyophilized to prepare a 10 mg/mL solution. The solution was filtered using a MF-Millipore 0.22 µm membrane (Merck, Darmstadt, Germany) before injecting to the UPLC system. The CSOSs were separated and analyzed using the hydrophilic interaction liquid chromatography (HILIC) with the help of a Phenomenex (Tianjin, China) Luna column (50 × 2.00 mm, 3 μm, C18) running at 25 °C.

The mobile phase used for the UPLC-MS/MS analysis was a mixed solution of 5 mM ammonium acetate/acetonitrile (A) and 5 mM ammonium acetate /water (B). The high-pressure pump for the mobile phase was programmed at 92% of solution A and 8% of solution B in the first minute and then linearly changed to 60% of solution A and 40% of solution B in the last minute. The ﬂow rate of the mobile phase was set at 150 μL/min for the UPLC-MS/MS analysis. The MS analysis was performed under the negative ion mode at a capillary temperature of 275 °C. The spray voltage in the mass spectrometer was set at 4.2 kV and the ﬂow rate of the purified dry N_2_ was set at 40 L/min. All the samples were analyzed in a randomized order.

Data acquisition and bioinformatics analysis were performed using Thermo Fisher Scientific Xcalibur software (Waltham, MA, USA) and GlycResoft software as previously described [2, 5-7]. The parameters of the GlycReSoft software used for the CSOSs analysis were set as follows: minimum abundance, 1.0; minimum number of scans, 1; molecular weight lower boundary, 200 Da; molecular weight upper boundary, 4000 Da; mass shift, ammonium; match error, 5.0 ppm; grouping error, 80 ppm; adduct tolerance, 5.0 ppm; C, ΔHexA = 0-1; F, HexA = 0-10; G, HexNAc = 0-10; P, SO_3_ = 0-10; P-G = 0.

**1.7 Isolation of CS-degrading bacteria from the human fecal samples**

The CS-degrading bacteria in all the 23 human fecal samples were isolated and purified using the well-established enrichment culture method [3, 6, 8]. Briefly, the CS-degrading bacteria in the human gut microbiota were first enriched for 3 times using the VI medium that contained CS as the major carbon source. Then, the CS-degrading bacteria were isolated and purified using the streak plate method.

The 16S rRNA gene of the isolates were first amplified with universal primers 27F (AGAGTTTGATCMTGGCTCAG) and 1492R (GGTTACCTTGTTACGACTT) and then sequenced using the Thermo Fisher Scientific 3730XL DNA Analyzer (Waltham, MA, USA) from the Sangon Biotech (Shanghai, China). The taxonomic assignments of the isolated bacteria were performed using the EzBioCloud Database (https://www.ezbiocloud.net/) as previously described [9]. Phylogenetic tree analysis of the CS-degrading bacteria from the human gut microbiota was performed using the Molecular Evolutionary Genetics Analysis (MEGA) software (version 7.0.26) as previously described [10].

**1.8 Morphological studies of *B. salyersiae* CSP6**

Transmission electron microscope (TEM) analysis of the cell morphology of *B. salyersiae* CSP6 was conducted with the help of the Servicebio Technology (Wuhan, Hubei, China). Briefly, *B. salyersiae* CSP6 was cultured in liquid VI medium that contained CS as the major carbon source. The bacterial cells were collected at the exponential phase and the TEM images were captured using a HT7800 microscope from the Hitachi High-Tech (Shanghai, China). The electron accelerating voltage of the TEM was set at 80.0 kV to capture the image of the bacterial cells.

**1.9 Whole genome sequencing of** ***B. salyersiae* CSP6**

*B. salyersiae* CSP6 was grown in liquid VI medium that contained CS as the major carbon source. About 120 mL of the culture medium were collected at the exponential phase. The medium was centrifugated at 8,000 *g* for 15 minutes to collect the bacterial cells using a Beckman Coulter Microfuge 20 benchtop centrifuge (Brea, CA, USA). The genomic DNA of *B. salyersiae* CSP6 was extracted using the QIAamp DNA mini kit (Qiagen, Hilden, Germany). The concentration of the obtained DNA was determined using a Thermo Fisher Scientific NanoDrop 2000 spectrophotometer (Waltham, MA, USA). A total of 12.50 μg DNA was obtained and used for the sequencing analysis in the present study.

The whole genome of *B. salyersiae* CSP6 was sequenced on an Illumina HiSeq platform and an Oxford Nanopore Technologies (ONT) Nanopore PromethION platform (Oxford, Cambridge, UK) from the Majorbio Bio-Pharm Biotechnology (Shanghai, China). Bioinformatics analysis of the sequencing data, including clusters of orthologous genes (COG) function classification, Kyoto Encyclopedia of Genes and Genomes (KEGG) pathway analysis, and carbohydrate-active enzyme (CAZymes, http://www.cazy.org/) analysis were all conducted using the online bioinformatic tools from the Majorbio Cloud Platform (www.majorbio.com) as previously described [11].

**1.10 16S rRNA gene amplicon high-throughput sequencing and bioinformatics analysis**

Metagenomic DNA of the human gut microbiota was extracted from the fecal samples and the fermentation media using a QIAamp Power Fecal Pro DNA Kit (Qiagen, Hilden, Germany). The obtained DNA was checked for quality, and the V3 to V4 hypervariable regions of the 16S rRNA gene were specifically amplified using the universal primers 338F (ACTCCTACGGGAGGAGCAG) and 806R (GGACTACHVGGGTWTCTAAT) as previously described [1, 5, 6].

The 16S rRNA gene amplicons were sequenced on an Illumina PE300 platform (San Diego, CA, USA) from Majorbio Bio-Pharm Biotechnology (Shanghai, China). Bioinformatic analyses of the sequencing data, including linear discriminant analysis (LDA) effect size (LEfSe) analysis, Wilcoxon rank-sum test analysis, observed species analysis, Chao1 index analysis, Shannon index analysis, Venn diagram, principal components analysis (PCA), and heatmap analysis were all conducted using the online tools from the Majorbio Cloud Platform (www.majorbio.com) as previously described [11].

**1.11 Spent medium assay**

The spent medium assay was conducted using the method adopted from previous studies [12-15]. Briefly, *B. salyersiae* CSP6 was first cultured in the VI medium containing CS at a concentration of 8 g/L. After 48 hours, the fermentation medium was collected and *B. salyersiae* CSP6 was removed by centrifugation at 8,000 *g* for 15 minutes using a Beckman Coulter Microfuge 20 benchtop centrifuge (Brea, CA, USA). After that, the medium was filter-sterilized using a MF-Millipore 0.22 µm membrane (Merck, Darmstadt, Germany).

The spent medium (cell-free supernatant) containing CSOSs (mainly udp4) as the major carbon source was used to culture the second bacterium. Total carbohydrate analysis, TLC analysis, and UHPLC-MS/MS analysis were conducted to investigate the utilization of CSOSs (mainly udp4) by the second bacterium. *B. salyersiae* CSP6 was also inoculated to the spent medium to serve as a negative control for the experiments. The fermentation experiments of *B. salyersiae* CSP6, *B. stercoris* P22-5, and *B. stercoris* P22-28 were conducted with three biological replicates (n=3).

**1.12 Statistical analysis**

All results were expressed as the mean ± standard error of mean (SEM). The statistical analysis was performed using Student’s t-test from the GraphPad Prism 8.0.2 software (Boston, MA, USA). The results were considered statistically significant at *p* < 0.05. **p* < 0.05; ***p* < 0.01; ****p* < 0.001.

**References**

1. Fu T, Pan L, Shang Q, Yu G. Fermentation of alginate and its derivatives by different enterotypes of human gut microbiota: Towards personalized nutrition using enterotype-specific dietary fibers. Int J Biol Macromol. 2021;183:1649-1659.
2. Pan L, Ai X, Fu T, Ren L, Shang Q, Li G, Yu G. *In vitro* fermentation of hyaluronan by human gut microbiota: Changes in microbiota community and potential degradation mechanism. Carbohydr Polym. 2021;269:118313.
3. Shang Q, Yin Y, Zhu L, Li G, Yu G, Wang X. Degradation of chondroitin sulfate by the gut microbiota of Chinese individuals. Int J Biol Macromol. 2016;86:112-8.
4. Nielsen S.S, Phenol-sulfuric acid method for total carbohydrates, in: Food Analysis Laboratory Manual, Springer, 2010, pp. 47–53.
5. Pan L, Ma M, Wang Y, et al. Polyguluronate alleviates ulcerative colitis by targeting the gut commensal *Lactobacillus murinus* and its anti-inflammatory metabolites. Int J Biol Macromol. 2024; 257:128592.
6. Fu T, Wang Y, Ma M, Dai W, Pan L, Shang Q, Yu G. Isolation of alginate-degrading bacteria from the human gut microbiota and discovery of *Bacteroides xylanisolvens* AY11-1 as a novel anti-colitis probiotic bacterium. Nutrients. 2023;15(6):1352.
7. Wang M, Shajahan A, Pepi LE, Azadi P, Zaia J. Glycoproteomic sample processing, LC-MS, and data analysis using GlycReSoft. Curr Protoc. 2021;1(3):e84. doi:10.1002/cpz1.84
8. Tidjani Alou M, Naud S, Khelaifia S, Bonnet M, Lagier JC, Raoult D. State of the art in the culture of the human microbiota: new interests and strategies. Clin Microbiol Rev. 2020;34(1):e00129-19.
9. Yoon SH, Ha SM, Kwon S, et al. Introducing EzBioCloud: a taxonomically united database of 16S rRNA gene sequences and whole-genome assemblies. Int J Syst Evol Microbiol. 2017;67(5):1613-1617.
10. Kumar S, Stecher G, Tamura K. MEGA7: Molecular evolutionary genetics analysis version 7.0 for bigger datasets. Mol Biol Evol. 2016;33(7):1870-1874.
11. Ren Y, Yu G, Shi C, et al. Majorbio Cloud: A one‐stop, comprehensive bioinformatic platform for multiomics analyses. iMeta, 2022, 1(2): e12.
12. Weiss AS, Burrichter AG, Durai Raj AC, von Strempel A, Meng C, Kleigrewe K, Münch PC, Rössler L, Huber C, Eisenreich W, Jochum LM, Göing S, Jung K, Lincetto C, Hübner J, Marinos G, Zimmermann J, Kaleta C, Sanchez A, Stecher B. *In vitro* interaction network of a synthetic gut bacterial community. ISME J. 2022;16(4):1095-1109.
13. Deines P, Hammerschmidt K, Bosch TCG. Microbial species coexistence depends on the host environment. mBio. 2020;11(4):e00807-20.
14. Liu B, Garza DR, Gonze D, et al. Starvation responses impact interaction dynamics of human gut bacteria *Bacteroides thetaiotaomicron* and *Roseburia intestinalis*. ISME J. 2023;17(11):1940-1952.
15. Lax S, Abreu CI, Gore J. Higher temperatures generically favour slower-growing bacterial species in multispecies communities. Nat Ecol Evol. 2020;4(4):560-567.

**2. Supplementary Figures and Tables**


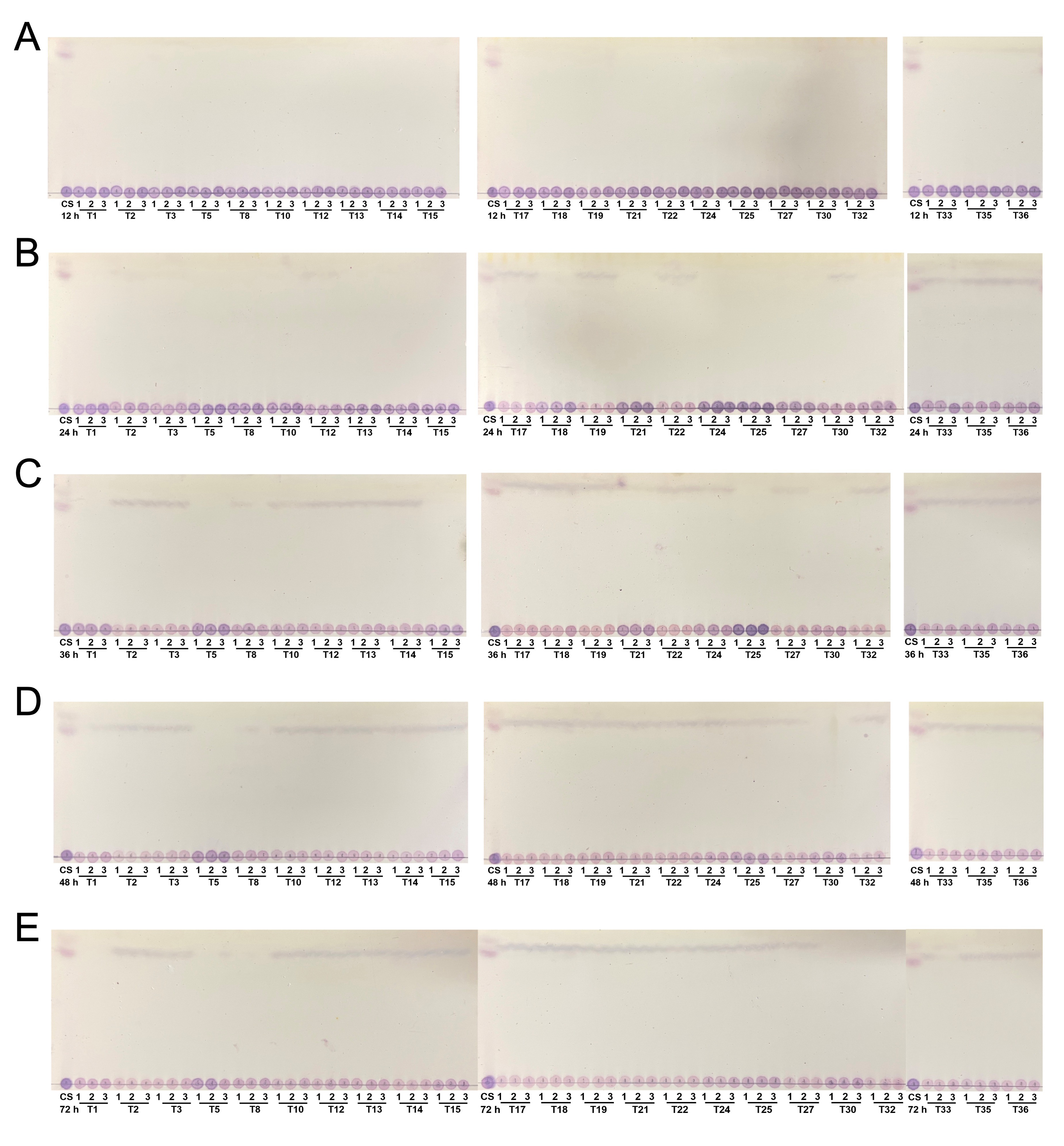


Figure S1. TLC showing the degradation of CS by the human gut microbiota. The degradation was monitored at 12 hours (A), 24 hours (B), 36 hours (C), 48 hours (D), and 72 hours (E).


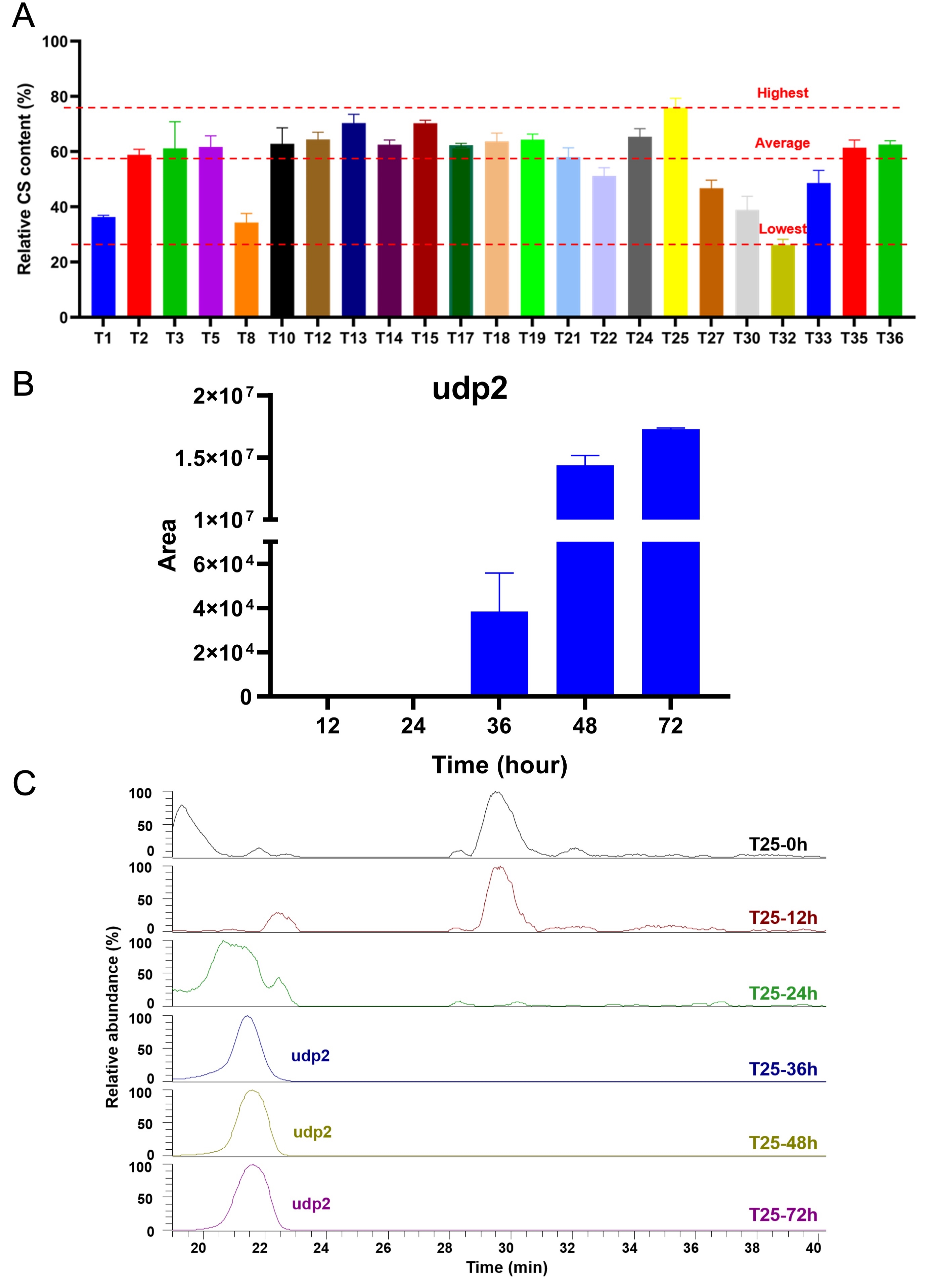


Figure S2. Degradation of CS by the human gut microbiota. Relative CS content in the culture medium at 72 hours (A). UPLC-MS/MS analysis of CSOSs in the culture medium of donor T25 (B). Total ion chromatograms showing the elution profiles of CSOSs in the culture medium of donor T25 at different time points (C).


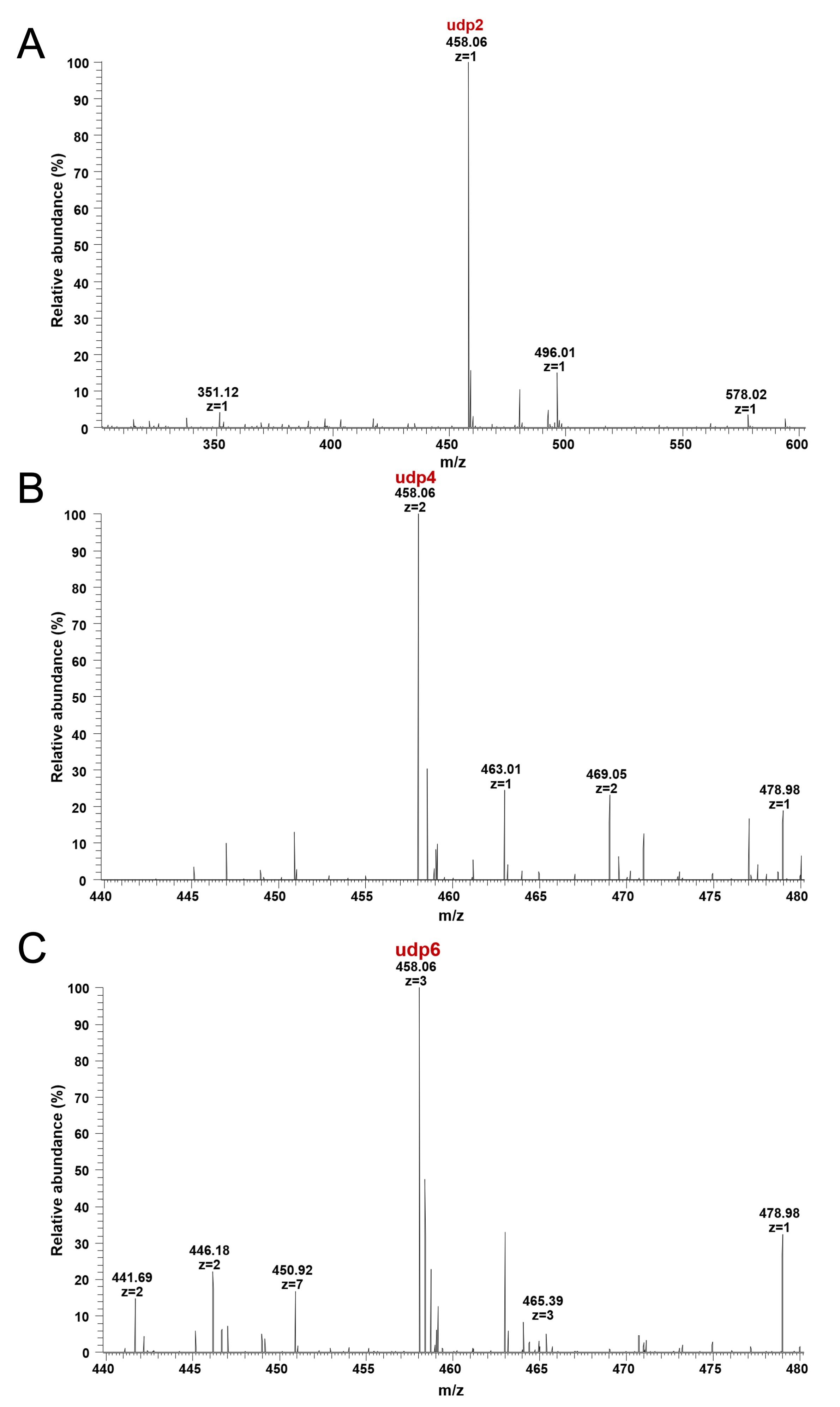


Figure S3. Mass spectrum showing the signals of udp2 (A), udp4 (B), and udp6 (C) according to their m/z ratios. The CSOSs, including udp2, udp4, and udp6 were produced in the culture medium as a result of CS degradation by the human gut microbiota.


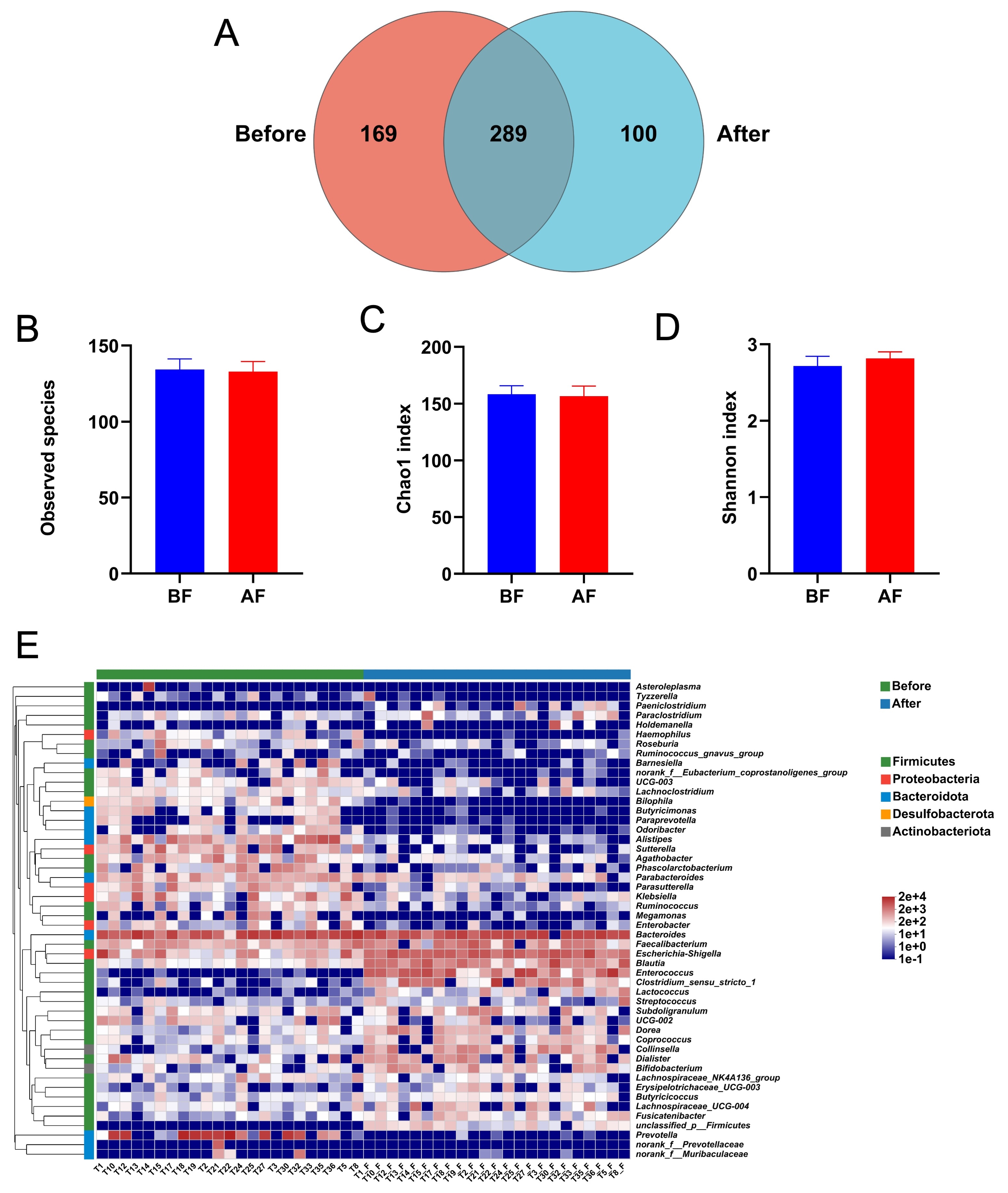


Figure S4. Changes in the structure of the human gut microbiota before and after fermentation. Venn diagram showing the differences of the operational taxonomic units (OTUs) (A). Observed species (B). Chao1 index (C). Shannon index (D). Heatmap of the abundance of gut bacteria at the genus level (E).


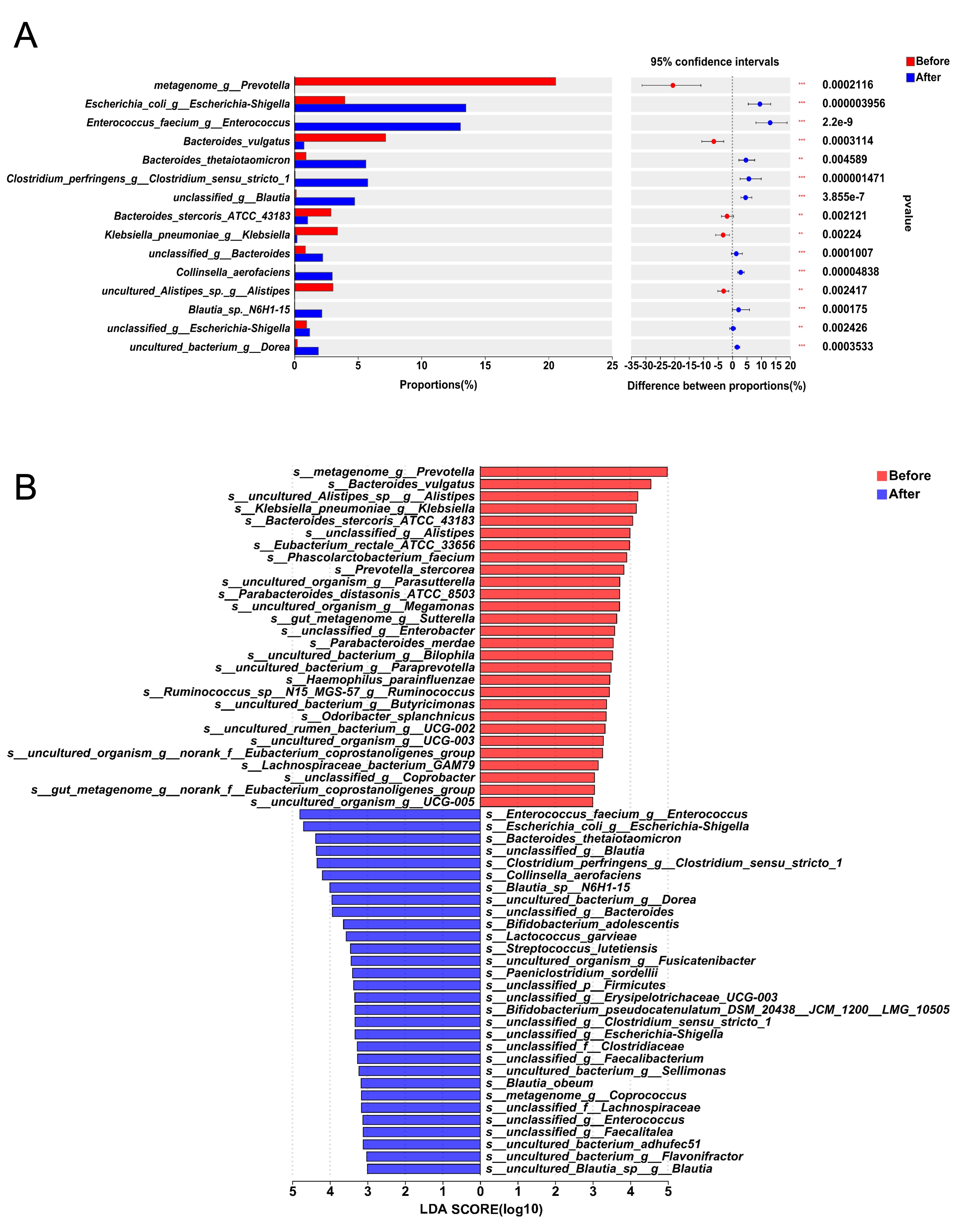


Figure S5. Differences in the composition of the human gut microbiota before and after fermentation. Wilcoxon rank-sum test analysis of the gut microbiota at the species level (A). Linear discriminant analysis (LDA) Effect Size (LEfSe) analysis of the gut microbiota at the species level (B). Only bacterial taxa with an LDA score of above 3.0 were listed.


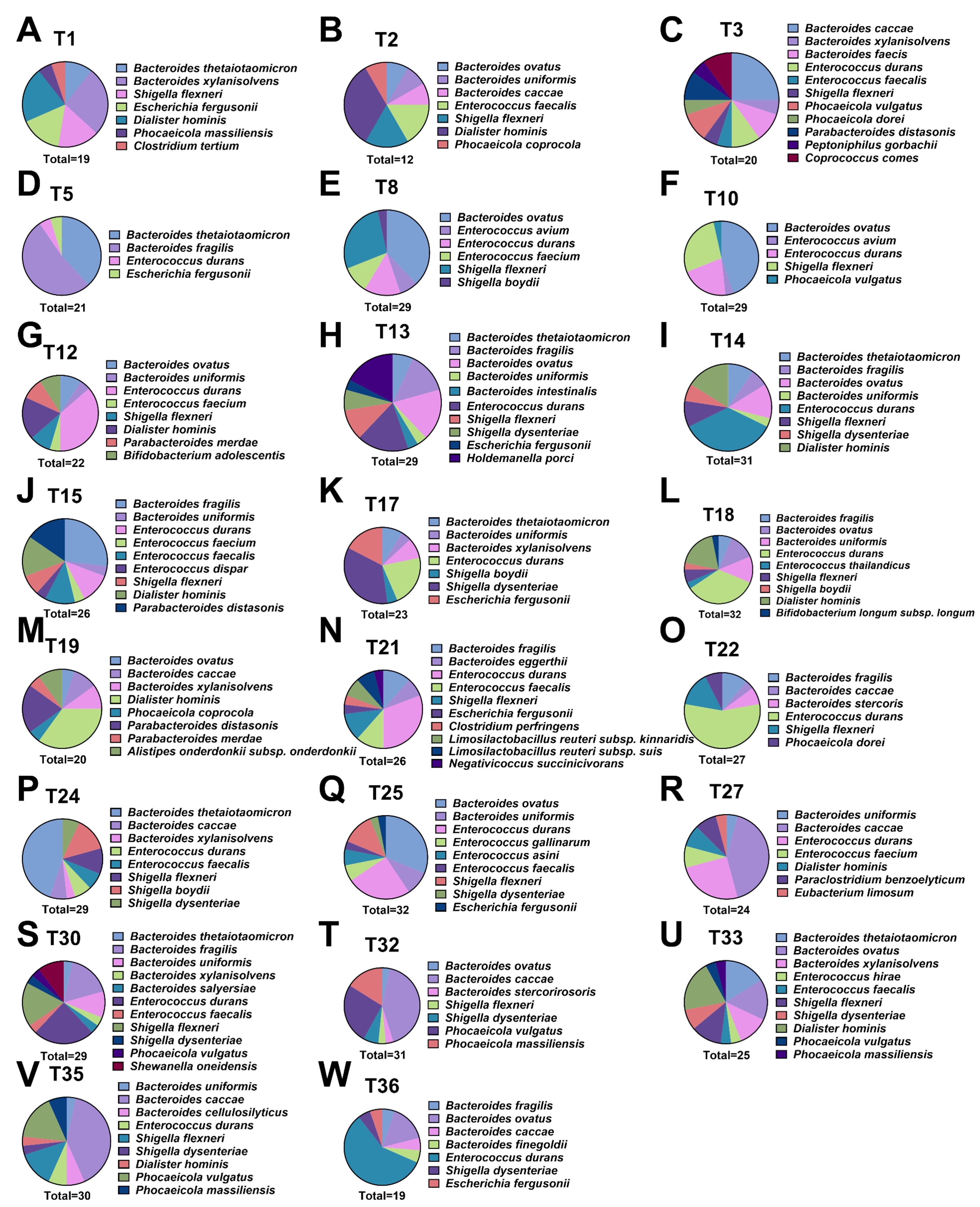


Figure S6. Isolation of CS-degrading bacteria from the human gut microbiota. Different species of bacteria were obtained from different human fecal samples (A-W).


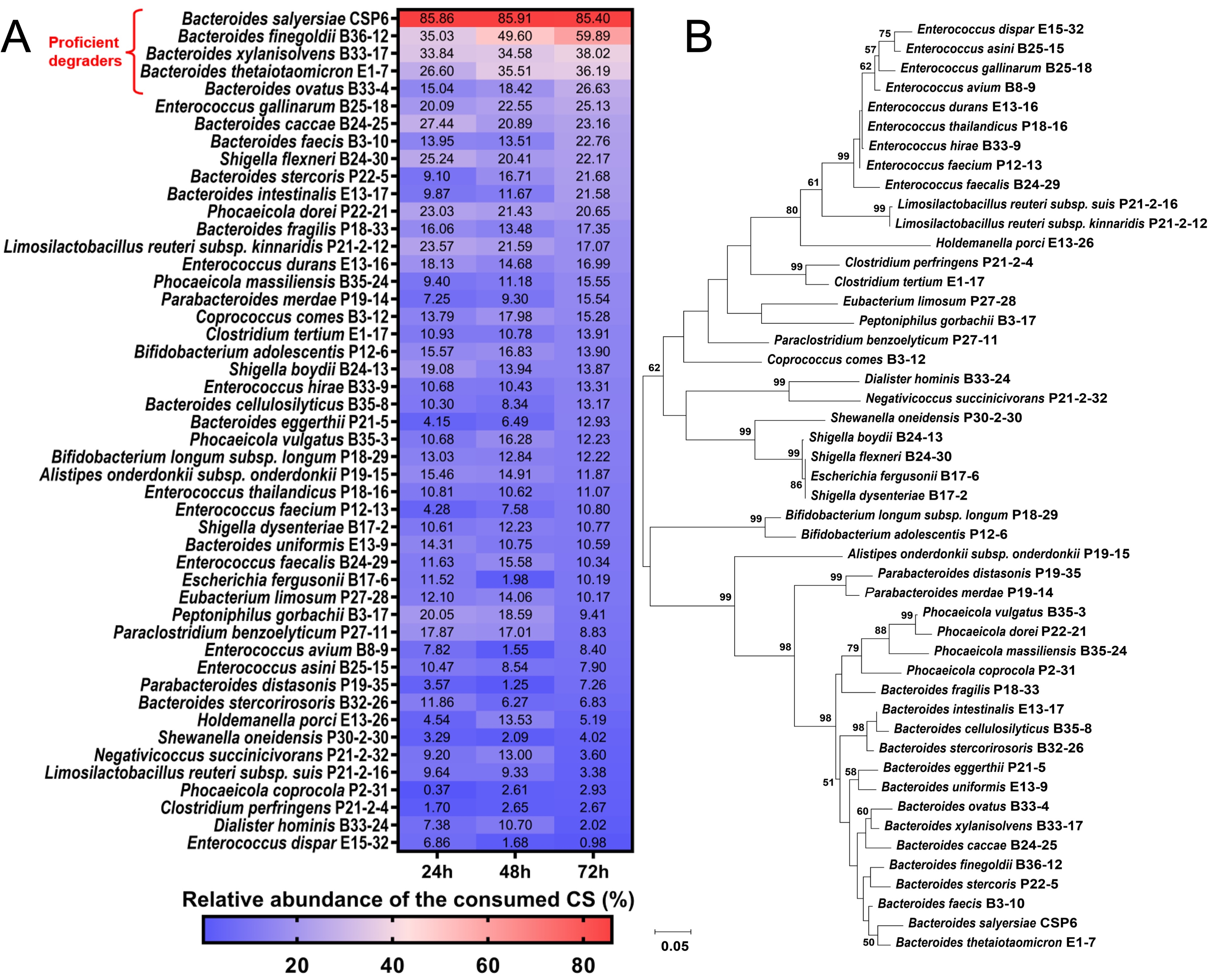


Figure S7. *B. salyersiae* CSP6 was identified as a potent bacterium for CS-degradation in the present study. Heatmap of the relative abundance of the consumed CS (A). Phylogenetic tree analysis of the CS-degrading bacteria based on the 16S rRNA gene (B).


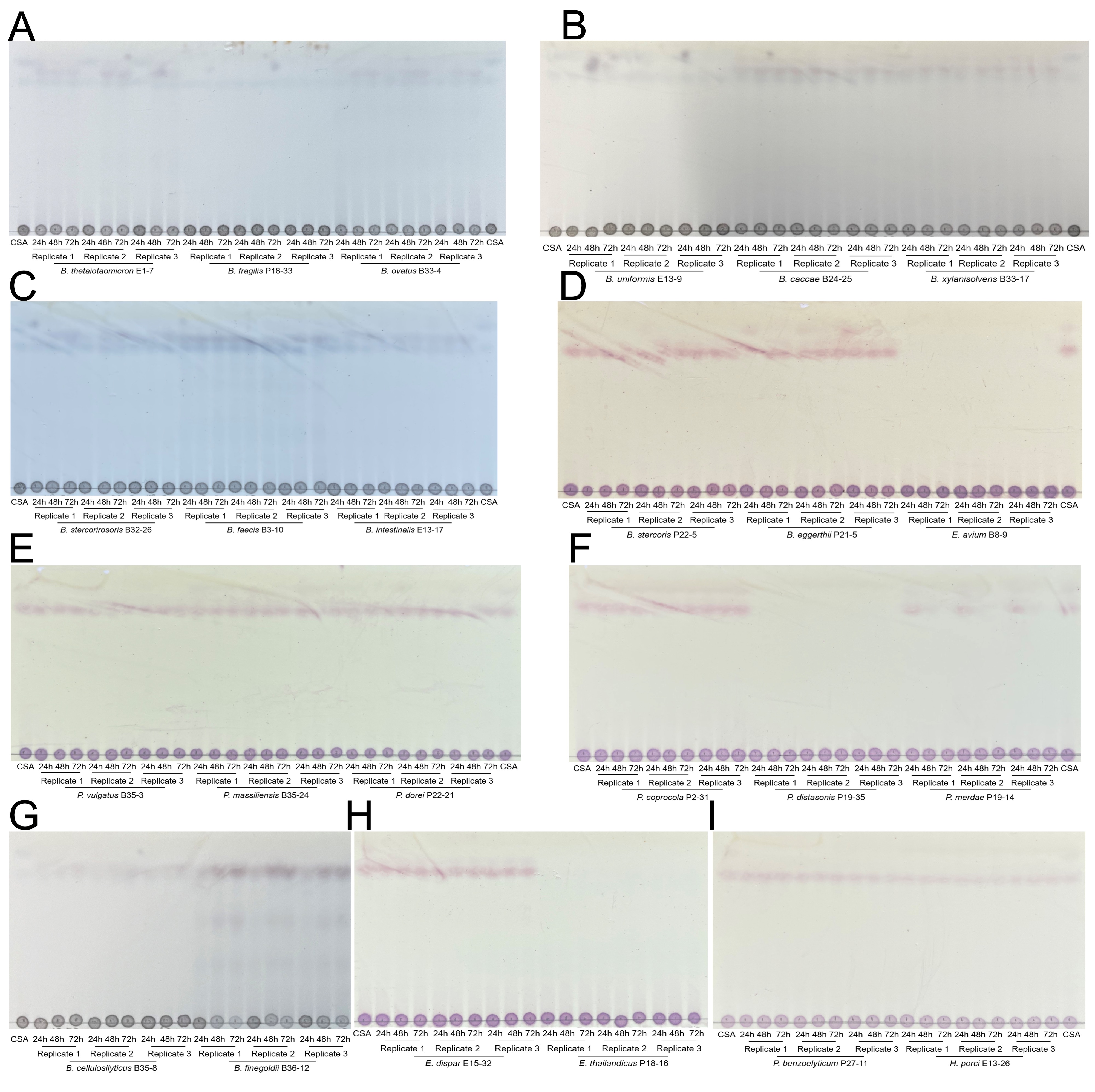


Figure S8. TLC showing the degradation of CS by different human fecal isolates. The results were presented from *B. thetaiotaomicron* E1-7 to *H. porci* E13-26 (A-I).


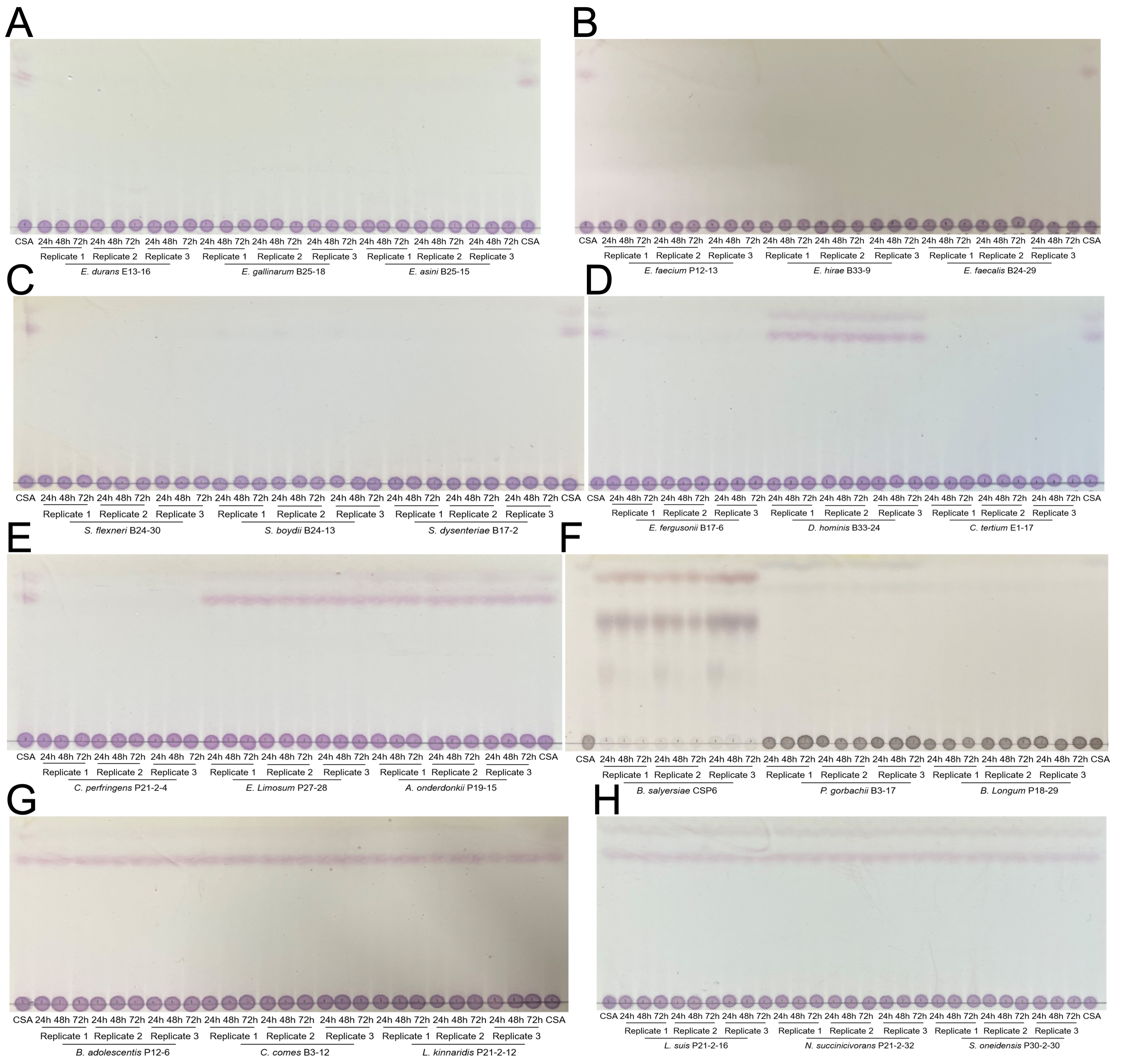


Figure S9. TLC showing the degradation of CS by different human fecal isolates. The results were presented from *E. durans* E13-16 to *S. oneidensis* P30-2-30 (A-H).


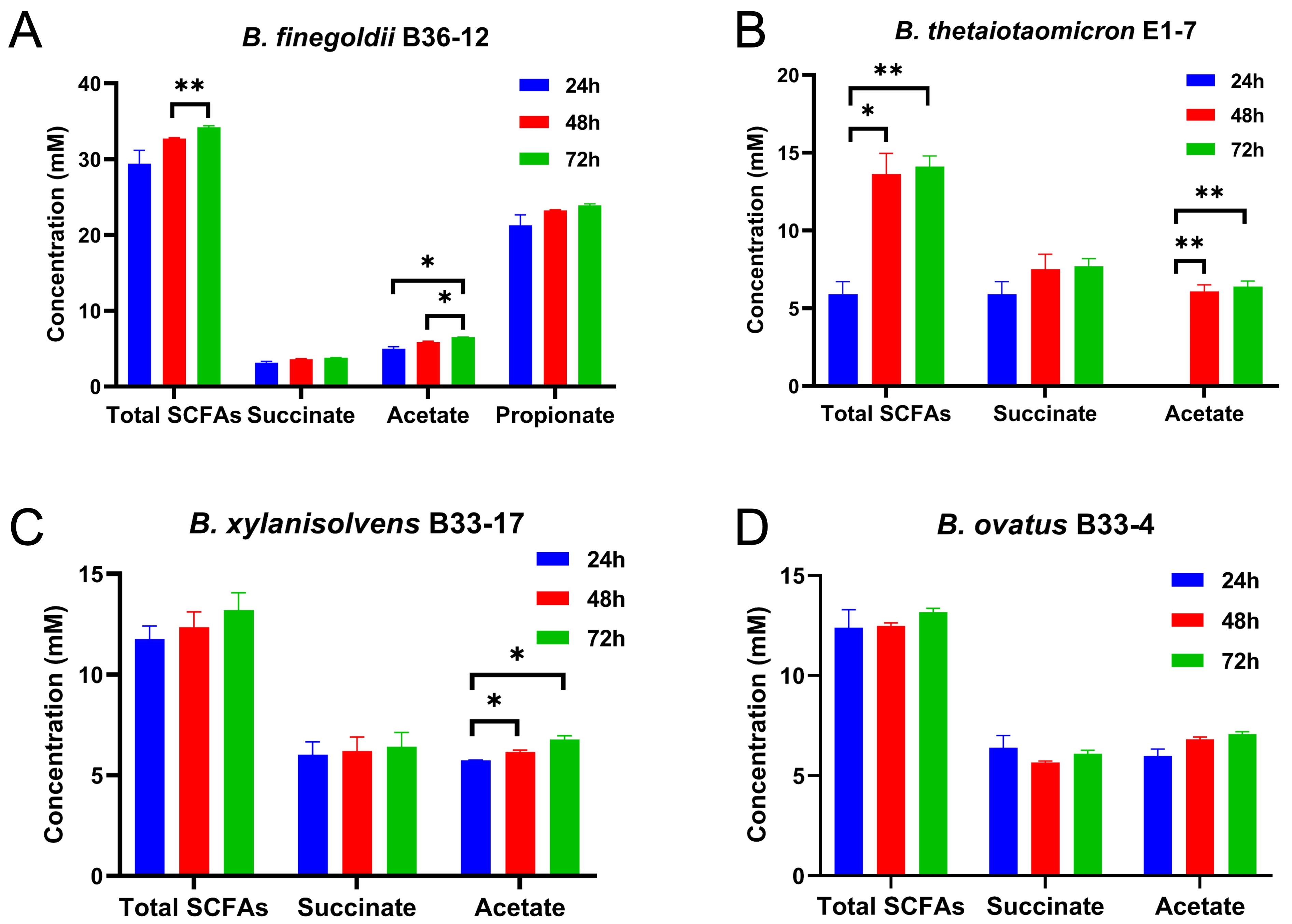


Figure S10. Degradation and fermentation of CS by *B. finegoldii* B36-12, *B. thetaiotaomicron* E1-7, *B. xylanisolvens* B33-17, and *B. ovatus* B33-4. Concentrations of different SCFAs in the culture medium of *B. finegoldii* B36-12 (A), *B. thetaiotaomicron* E1-7 (B), *B. xylanisolvens* B33-17 (C), and *B. ovatus* B33-4 (D). * *p* < 0.05; ** *p* < 0.01.


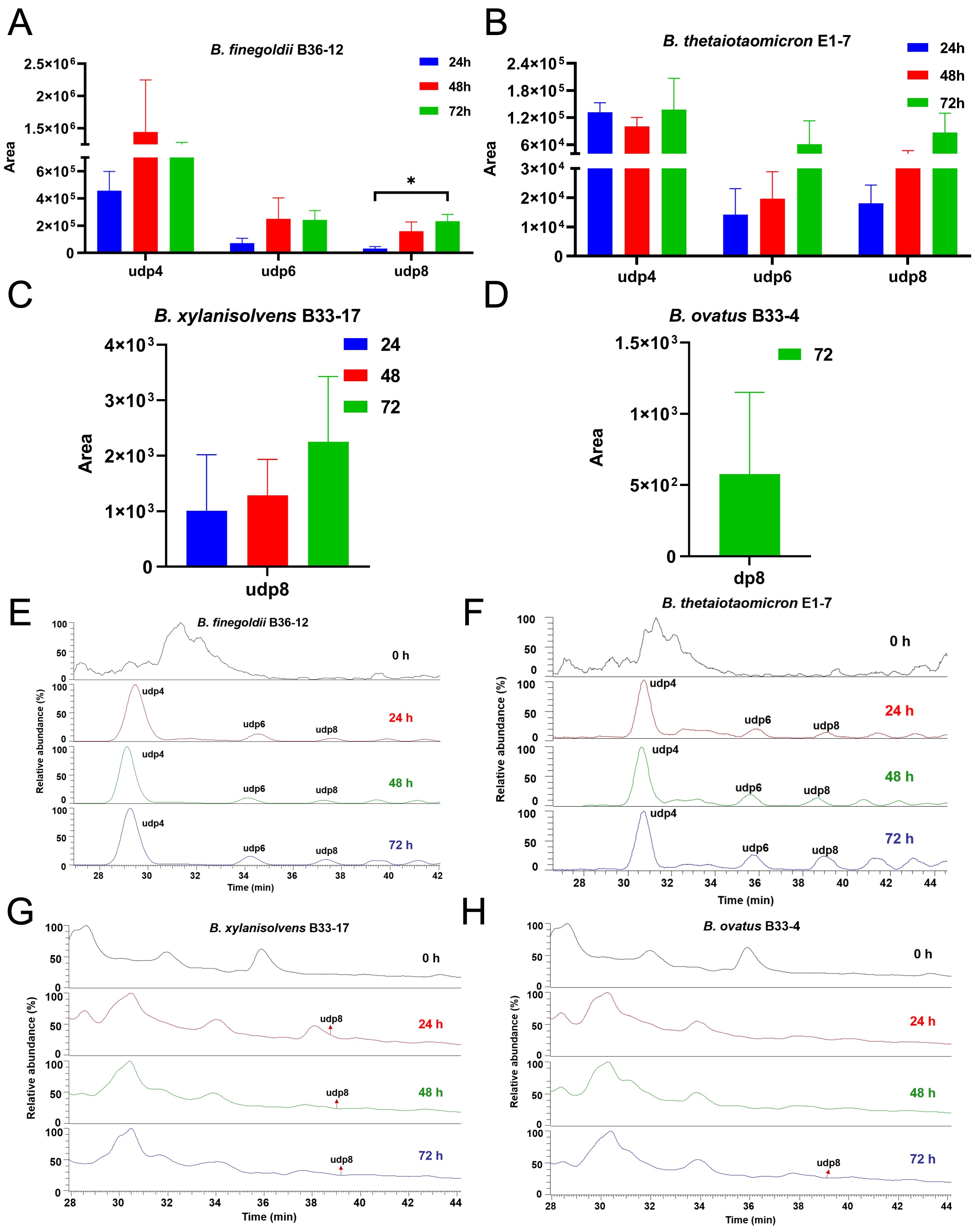


Figure S11. CS degradation by *B. finegoldii* B36-12, *B. thetaiotaomicron* E1-7, *B. xylanisolvens* B33-17, and *B. ovatus* B33-4. UPLC-MS/MS analysis of CSOSs produced by *B. finegoldii* B36-12 (A), *B. thetaiotaomicron* E1-7 (B), *B. xylanisolvens* B33-17 (C), and *B. ovatus* B33-4 (D). Total ion chromatograms showing the elution profiles of CSOSs in the culture medium of *B. finegoldii* B36-12 (E), *B. thetaiotaomicron* E1-7 (F), *B. xylanisolvens* B33-17 (G), and *B. ovatus* B33-4 (H) at different time points. * *p* < 0.05.


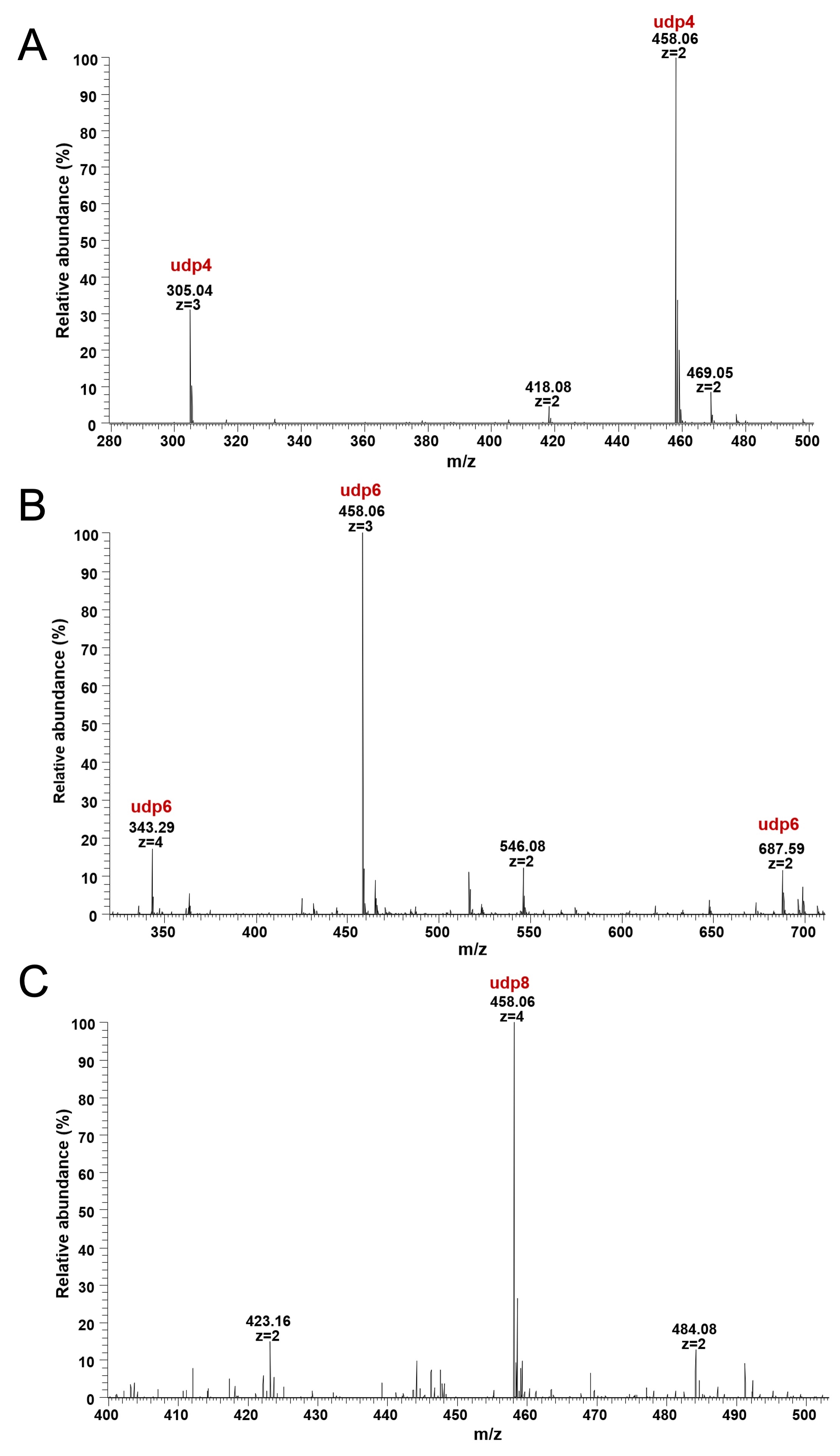


Figure S12. Mass spectrum showing the signals of udp4 (A), udp6 (B), and udp8 (C) according to their m/z ratios. The CSOSs, including udp4, udp6, and udp8 were produced in the culture medium as a result of CS degradation by *B. salyersiae* CSP6, *B. finegoldii* B36-12, *B. xylanisolvens* B33-17, *B. thetaiotaomicron* E1-7, and *B. ovatus* B33-4.


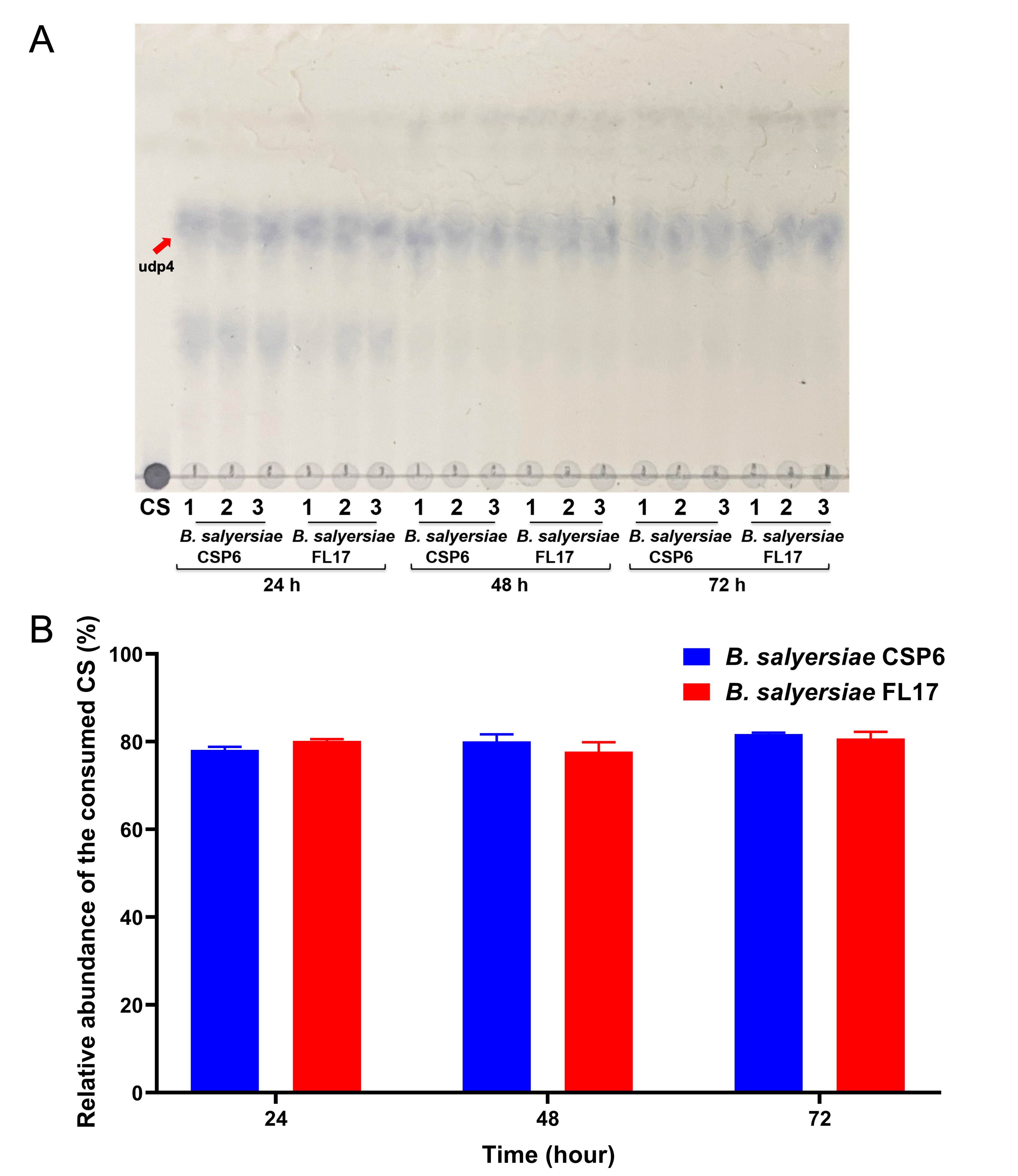


Figure S13. Degradation of CS by different strains of *B. salyersiae*. TLC showing the degradation of CS by *B. salyersiae* CSP6 and *B. salyersiae* FL17 (A). Relative carbohydrate content in the culture medium at different time points (B). *B. salyersiae* FL17 was previously isolated from the fecal sample of a healthy individual. This individual has not participated in the present study.


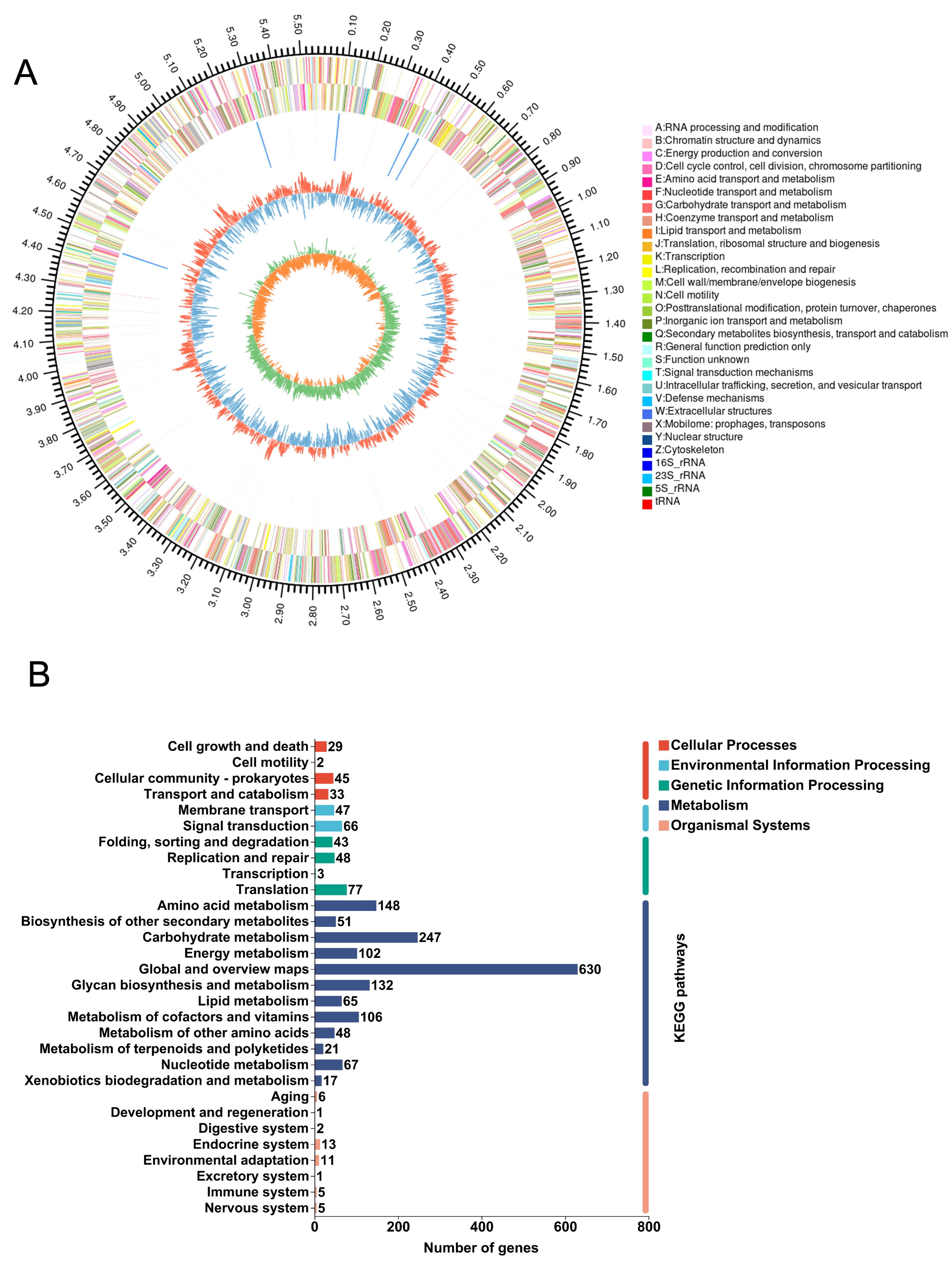


Figure S14. Genome analysis of *B. salyersiae* CSP6. COG function classification (A). KEGG pathway analysis (B).


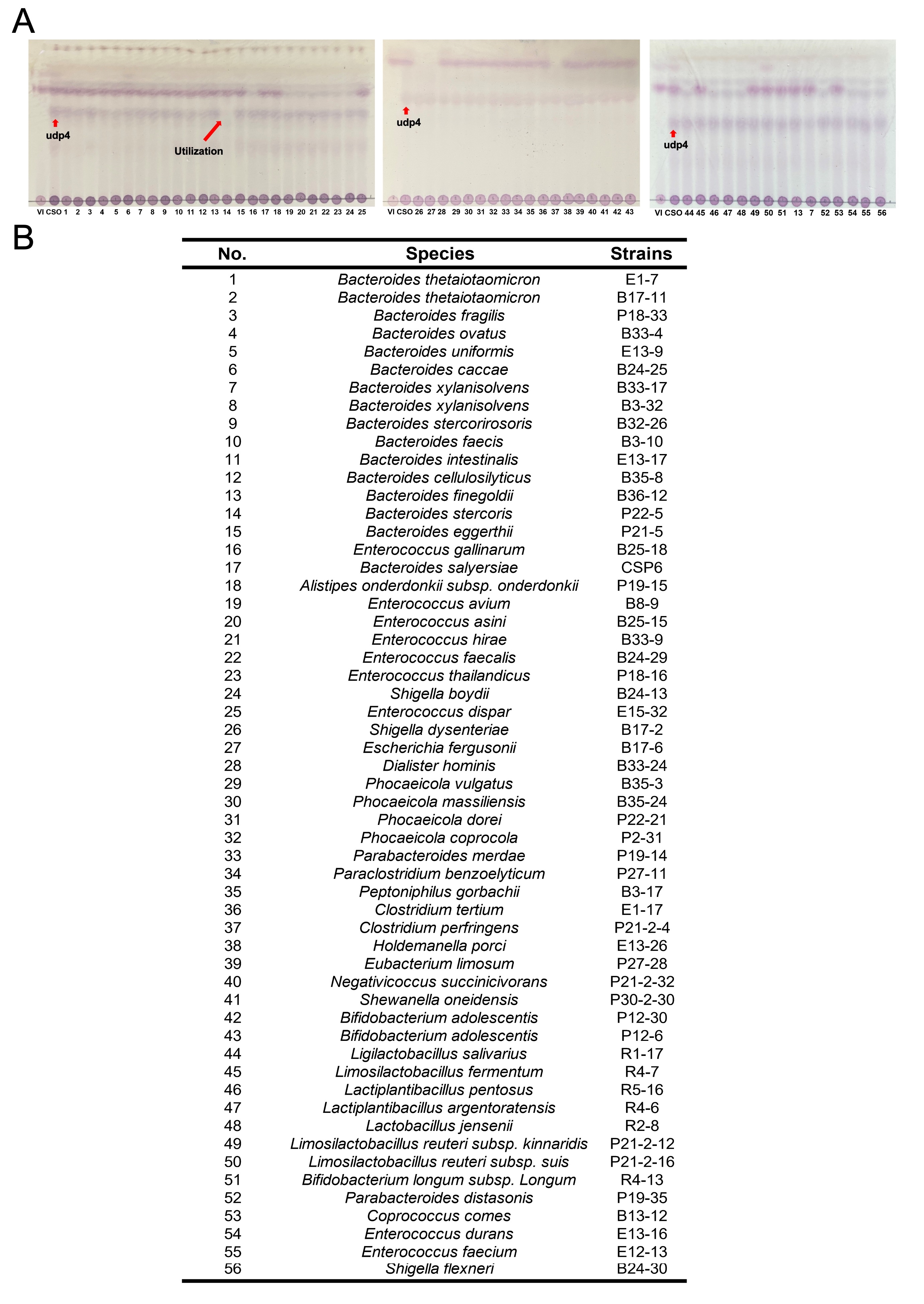


Figure S15. Screening of candidate bacteria that could utilize udp4 using the spent medium assay. TLC showing the utilization of udp4 by different human gut bacteria. (A). List of the tested bacteria (B).


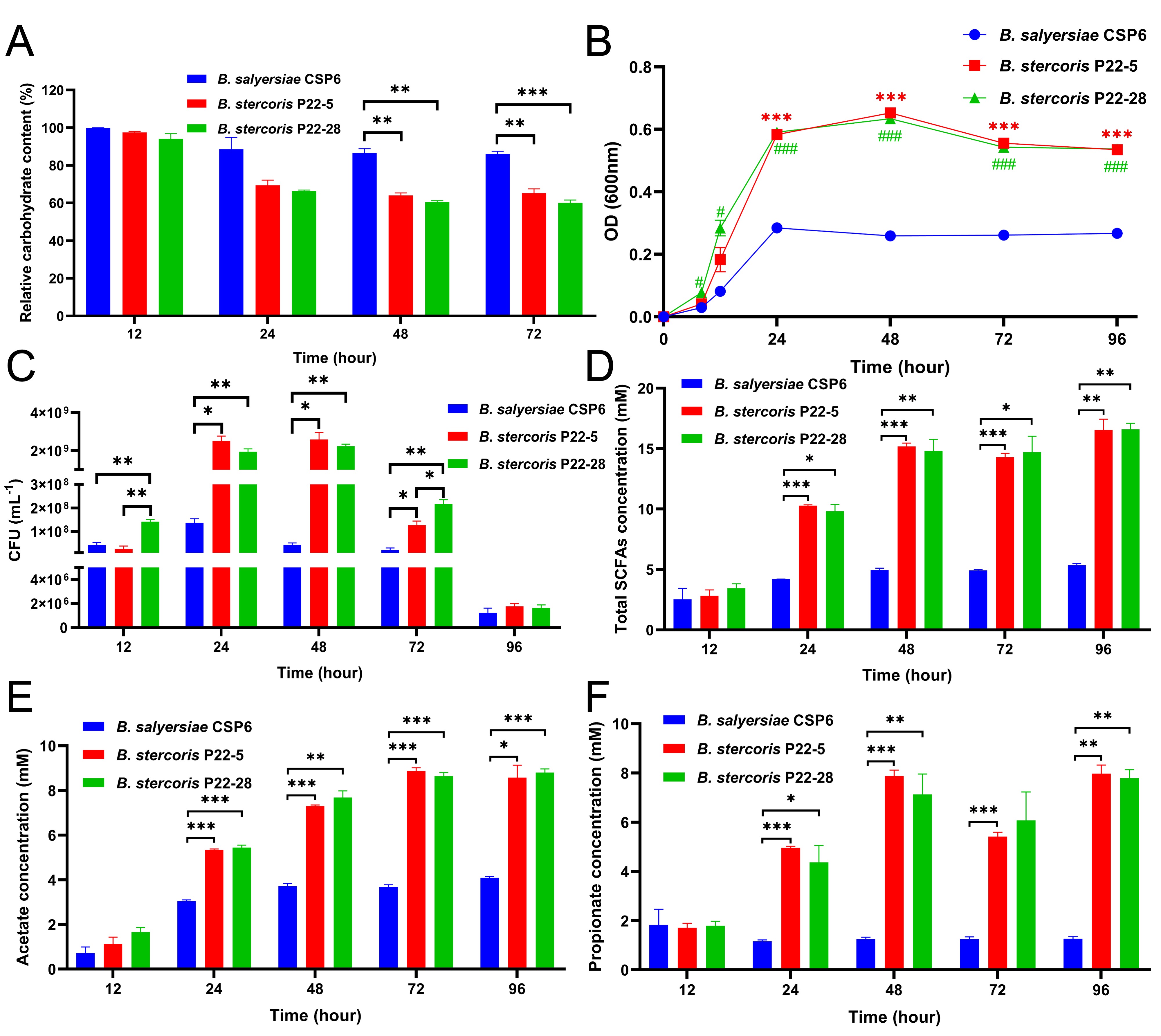


Figure S16. Cross-feeding interactions between *B. salyersiae* and *B. stercoris* identified using the spent medium assay. Relative carbohydrate content in the culture medium (A). Growth curve (B) and CFU analysis (C). Concentrations of total SCFAs (D), acetate (E), and propionate (F) in the culture medium of *B. salyersiae* and *B. stercoris*. * *p* < 0.05; ** *p* < 0.01; *** *p* < 0.001.


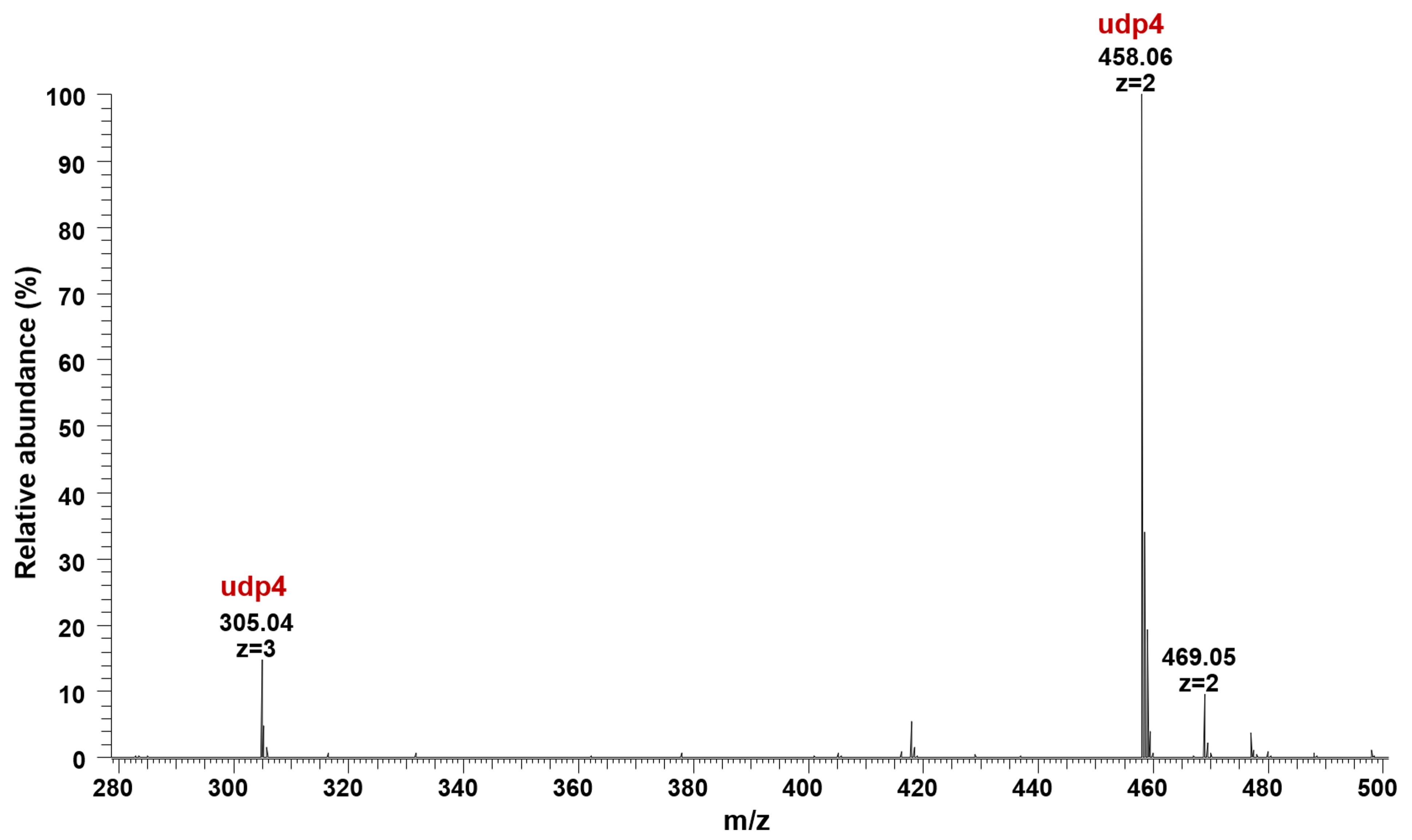


Figure S17. Mass spectrum showing the signal of udp4 according to the m/z ratio. The udp4 concentration in the spent medium was analyzed using UPLC-MS/MS.

Table S1 Summary of CS-degrading bacteria isolated from the human fecal samples.


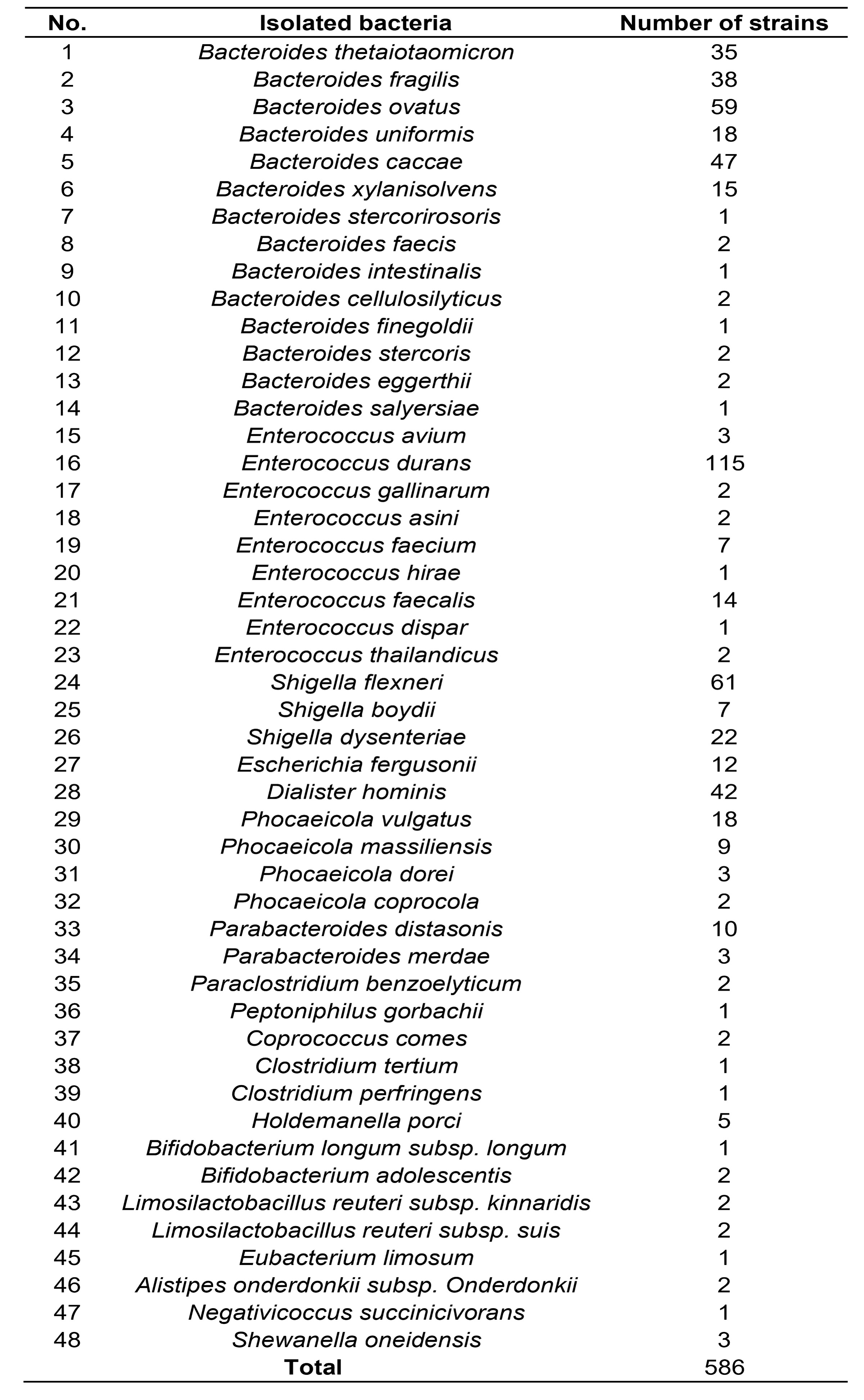


Table S2 Summary of the potential enzymes for CS degradation in *B. salyersiae* CSP6 based on the genomic analysis.


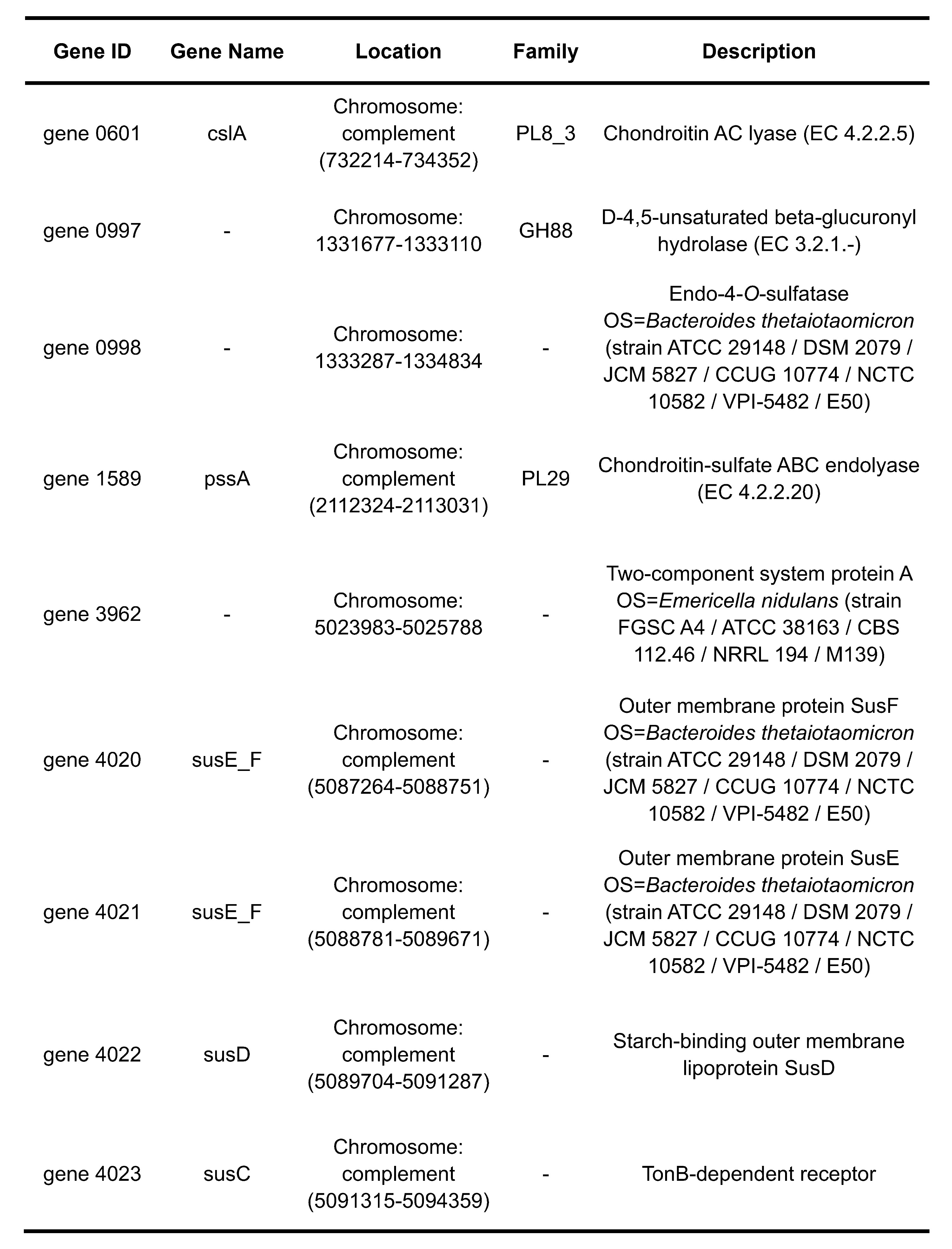

Supplement: Supplementary file 2 — Additional file 1: Figure S1. TLC showing the degradation of CS by the human gut microbiota. The degradation was monitored at 12 hours (A), 24 hours (B), 36 hours (C), 48 hours (D), and 72 hours (E). Figure S2. Degradation of CS by the human gut microbiota. Relative CS content in the culture medium at 72 hours (A). UPLC-MS/MS analysis of CSOSs in the culture medium of donor T25 (B). Total ion chromatograms showing the elution profiles of CSOSs in the culture medium of donor T25 at different time points (C). Figure S3. Mass spectrum showing the signals of udp2 (A), udp4 (B), and udp6 (C) according to their m/z ratios. The CSOSs, including udp2, udp4, and udp6 were produced in the culture medium as a result of CS degradation by the human gut microbiota. Figure S4. Changes in the structure of the human gut microbiota before and after fermentation. Venn diagram showing the differences of the operational taxonomic units (OTUs) (A). Observed species (B). Chao1 index (C). Shannon index (D). Heatmap of the abundance of gut bacteria at the genus level (E). Figure S5. Differences in the composition of the human gut microbiota before and after fermentation. Wilcoxon rank-sum test analysis of the gut microbiota at the species level (A). Linear discriminant analysis (LDA) Effect Size (LEfSe) analysis of the gut microbiota at the species level (B). Only bacterial taxa with an LDA score of above 3.0 were listed. Figure S6. Isolation of CS-degrading bacteria from the human gut microbiota. Different species of bacteria were obtained from different human fecal samples (A-W). Figure S7. B. salyersiae CSP6 was identified as a potent bacterium for CS-degradation in the present study. Heatmap of the relative abundance of the consumed CS (A). Phylogenetic tree analysis of the CS-degrading bacteria based on the 16S rRNA gene (B). Figure S8. TLC showing the degradation of CS by different human fecal isolates. The results were presented from B. thetaiotaomicron E1-7 to H. porci E13-26 (A-I). [file 40168_2024_1768_MOESM1_ESM.zip › Supplementary Methods, Materials, Figures, and Tables.docx]
